# Pharmacokinetics and safety of TBAJ-876, a novel antimycobacterial diarylquinoline, in healthy subjects

**DOI:** 10.1128/aac.00613-24

**Published:** 2024-08-28

**Authors:** Antonio Lombardi, Fran Pappas, Jerry Nedelman, Dean Hickman, Sarah Jaw-Tsai, Morounfolu Olugbosi, Paul Bruinenberg, Maria Beumont, Eugene Sun

**Affiliations:** 1 Global Alliance for TB Drug Development, New York, New York, USA; 2 Sarah Jaw-Tsai Consulting Services, San Francisco, California, USA; 3 Vast Therapeutics, Durham, North Carolina, USA; St. George's, University of London, London, United Kingdom

**Keywords:** TBAJ-876, diarylquinoline, tuberculosis, pharmacokinetics, phase 1, antimicrobial safety

## Abstract

TBAJ-876, a second-generation diarylquinoline with greater antimycobacterial activity and a potentially better safety profile compared with bedaquiline, is under development for the treatment of drug-susceptible and drug-resistant tuberculosis (TB). A phase 1, first-in-human study of TBAJ-876, comprising a single-ascending dose (SAD) part including a food effect cohort, a multiple-ascending dose (MAD) part, and a relative bioavailability part of tablets versus oral suspension, was conducted on 137 healthy adults. A drug–drug interaction study was conducted on 28 healthy adults to evaluate the effects of TBAJ-876 on a cytochrome P450 3A4 substrate (midazolam) and a P-glycoprotein substrate (digoxin). TBAJ-876 was well-tolerated at single doses up to 800 mg and multiple doses up to 200 mg for 14 days. No deaths or serious adverse events occurred. No episodes of clinically significant prolongation of the QTc interval were observed. TBAJ-876 exposures were dose proportional in the SAD and MAD studies. TBAJ-876 exhibited multicompartmental pharmacokinetics (PK) with a long terminal half-life yielding quantifiable concentrations up to the longest follow-up of 10 weeks after a single dose and resulting in accumulation with multiple dosing. In the fed state, TBAJ-876 exposures approximately doubled with the tablet formulation, whereas M3 metabolite exposures decreased by approximately 20%. The relative bioavailability of TBAJ-876 was similar between tablets and the oral suspension at 100-mg doses. With co-administration of TBAJ-876, the AUC_0-inf_ of midazolam was unchanged and the C_max_ was reduced by 14%; the AUC_0-last_ of digoxin was increased by 51%, and the C_max_ was increased by 18%. These results support further investigation of TBAJ-876 for the treatment of tuberculosis.

## INTRODUCTION

Despite progress in diagnosis and treatment, tuberculosis (TB) remains the leading cause of death due to infections in the world. The estimated yearly incidence of TB is approximately 10 million per year, with over 1 million people dying from the disease (1). Current treatment regimens for drug-susceptible TB (DS-TB) are efficacious but lengthy, which may lead to high rates of non-adherence, resulting in unfavorable outcomes and development of drug resistance (2).

TBAJ-876 is a novel oral diarylquinoline (DARQ) antibiotic under development for the treatment of both DS- and drug-resistant (DR-) TB. TBAJ-876 belongs to the same drug class as bedaquiline (BDQ), the first approved DARQ. Both inhibit ATP synthase, an enzyme essential for the generation of energy in TB mycobacteria. Although BDQ has become an important component of combination regimens for the treatment of multidrug-resistant (MDR) TB (3), it has a number of safety issues, and resistance to BDQ is emerging (4, 5). Compared with BDQ, TBAJ-876 is at least tenfold more potent in MIC assays against diverse strains of *Mycobacterium tuberculosis*, including strains harboring *Rv0678* mutants that confer resistance to BDQ (6). Its primary active metabolite, N-desmethyl-TBAJ-876 (M3), analogous to BDQ’s N-desmethyl metabolite M2, is similarly at least tenfold more potent than BDQ’s M2 (6). In a murine model of TB, TBAJ-876 demonstrated superior efficacy as monotherapy and within regimens compared to bedaquiline (7), including against mice infected with the *Rv0768* mutant (8). Nonclinical safety evaluations demonstrated acceptable toxicologic and tolerability profiles in rats and dogs. Importantly, TBAJ-876 and two circulating metabolites (M3 and the N-didesmethyl M2) had reduced QTc risk compared with BDQ and its corresponding metabolites. Based on these preclinical data, TBAJ-876 may have an advantageous efficacy and safety profile when compared with BDQ.

In this study, we present the results of CL-001, a three-part, Phase 1, first-in-human (FIH) study of the TBAJ-876 oral suspension in healthy volunteer adults comprising a single-ascending dose (SAD) part with a food-effect cohort, a multiple-ascending dose (MAD) part, and a relative bioavailability (rBA) part comparing two strengths of TBAJ-876 tablets with the oral suspension. We also present the results of CL-002, a drug–drug interaction (DDI) study in healthy adults evaluating the effects of TBAJ-876 on a cytochrome P450 3A4 (CYP3A4) substrate (midazolam) and a P-glycoprotein (P-gp) substrate (digoxin). *In vitro* data on TBAJ-876 demonstrated the potential for induction of CYP3A4 and inhibition of P-gp. The DDI study was planned in anticipation of a phase 2 study including patients with TB and HIV co-infection receiving tenofovir, a P-gp substrate, and dolutegravir, a P-gp and CYP3A4 substrate.

The data from these Phase 1 studies, together with the data from preclinical studies, provide the foundation for advancing TBAJ-876 as a next-generation DARQ with potentially improved safety and efficacy, including activity against BDQ-resistant TB.

## RESULTS

### Participants

A total of 137 healthy adult subjects were enrolled at a single site in CL-001 (SAD/MAD/rBA study) and were included in the safety analysis set. The pharmacokinetic (PK) analysis set included only subjects on active treatment, *n* = 112. The SAD part enrolled 68 subjects (55 on active; 13 on placebo). The MAD part enrolled 39 subjects (27 on active; 12 on placebo). The rBA part enrolled 30 subjects (all on active). In the SAD part, six subjects terminated study participation early (two for protocol non-compliance, two withdrew consent, and two were lost to follow-up). In the MAD part, three subjects terminated study participation early (two withdrew consent and one was withdrawn by the investigator due to COVID-19). In the rBA part, three subjects terminated study participation early (one tested positive for cotinine, one withdrew consent, and one was lost to follow-up).

In CL-002 (DDI study), 28 healthy adult subjects were enrolled at a single site (all on active) and included in the safety analysis set, of whom two were terminated early (one withdrew consent and one by the investigator’s judgment).

The demographic characteristics of the participants by the study and dose group are shown in Table 1.

**TABLE 1:** Participant demographics, CL-001 and CL-002

Group		Age (years)		Weight (kg)		Female	Hispanic or Latino^*a*^	White^*b*^	Black or African American^*b*^
	n	Mean	Range	Mean	Range	N (%)	N (%)	%	%
CL-001: Total	68	35.3	19–50	74.97	52–100	38 (55.9)	45 (66.2)	46 (67.6)	17 (25.0)
CL-001: SAD +Food effect									
Placebo fasted	13	36.1	24–50	73.85	57–93	5 (38.5)	11 (84.6)	10 (76.9)	2 (15.4)
10 mg fasted	6	41.2	29–48	73.27	65–80	3 (50.0)	5 (83.3)	4 (66.7)	1 (16.7)
25 mg fasted	6	39.8	24–50	76.58	58–99	3 (50.0)	3 (50)	2 (33.3)	3 (50)
50 mg fasted	6	34.0	26–45	79.33	63–98	3 (50.0)	5 (83.3)	5 (83.3)	1 (16.7)
100 mg susp.fasted	9	33.3	20–45	74.12	58–91	7 (77.8)	5 (55.6)	6 (66.7)	3 (33.3)
100 mg susp. fed	10	35.6	26–46	80.56	61–100	4 (40.0)	7 (70)	9 (90)	1 (10)
200 mg fasted	6	34.7	19–45	84.27	69–99	3 (50.0)	3 (50)	4 (66.7)	2 (33.3)
400 mg fasted	6	33.0	21–50	67.62	54–91	4 (66.7)	4 (66.7)	4 (66.7)	2 (33.3)
800 mg fasted	6	29.5	22–42	63.15	52–84	6 (100)	2 (33.3)	2 (33.3)	2 (33.3)
CL-001: Relative bioavailability of the tablet									
1 × 100 mg tab.fasted	10	36.8	19–50	78.03	58–94	3 (30.0)	7 (70)	7 (70)	2 (20)
4 × 25 mg tab. fasted	10	34.7	26–47	79.27	61–93	1 (10.0)	5 (50)	7 (70)	3 (30)
1 × 100 mg tab. fed	10	38.8	32–48	73.62	57–88	3 (30.0)	3 (30)	2 (20)	7 (70)
CL-001: MAD	39	35.2	19–48	74.56	54–115	22 (56.4)	18 (46.2)	26 (66.7)	11 (28.2)
Placebo fed	12	36.8	27–48	71.08	54–82	9 (75.0)	5 (41.7)	6 (50.0)	5 (41.7)
25 mg fed	9	35.7	24–46	75.13	58–93	3 (33.3)	3 (33.3)	5 (55.6)	3 (33.3)
75 mg fed	9	32.4	24–41	75.23	65–90	5 (55.6)	4 (44.4)	7 (77.8)	2 (22.2)
200 mg fed	9	35.3	19–48	77.93	56–115	5 (55.6)	6 (66.7)	8 (88.9)	1 (11.1)
CL-002 fed	28	37.1	20–55	78.2	49–153	14 (50)	8 (28.6)	8 (28.6)	20 (71.4)

^*a*^ “Hispanic or Latino” is a category of ethnicity; the complement is “Not Hispanic or Latino.”

^*b*^ “White” and “Black or African American” are categories of race; other categories were Asian, American Indian or Alaskan Native, Native Hawaiian or Other Pacific Islander, and Multiple Race, for which the total counts were 0, 2 (2.9%), 0, and 3 (4.4%), respectively, in CL-001.

### Pharmacokinetics

Concentrations of the parent, TBAJ-876, and the two circulating metabolites, M2 and M3, were measured in CL-001 and CL-002. Because of M2’s low exposures, the focus is on the parent and M3. Notably, in preclinical toxicology species, such as rats and dogs, M3 exposures were, respectively, similar to and greater than those of the parent, whereas M2 exposures were only about 20% that of the parent (internal data).

#### CL-001: Single-dose PK

**Fig 1:**
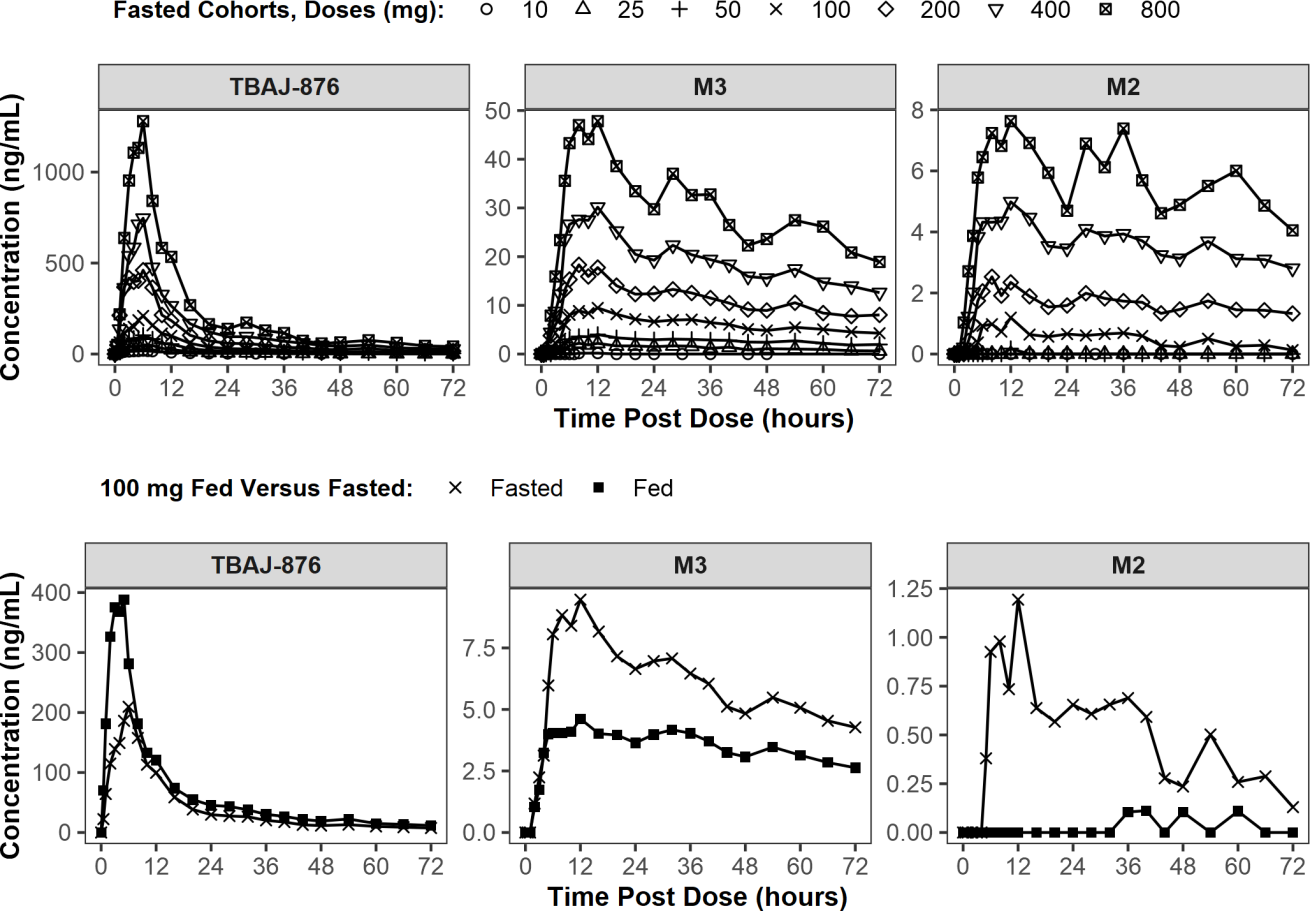
Mean plasma concentrations over time of TBAJ-876, M3, and M2 in the SAD part of CL-001. Top: after escalating, single, fasted doses of TBAJ-876 administered as an oral suspension. Bottom: as single doses of 100 mg of TBAJ-876 administered as an oral suspension in fasted and fed states.

**Fig 2:**
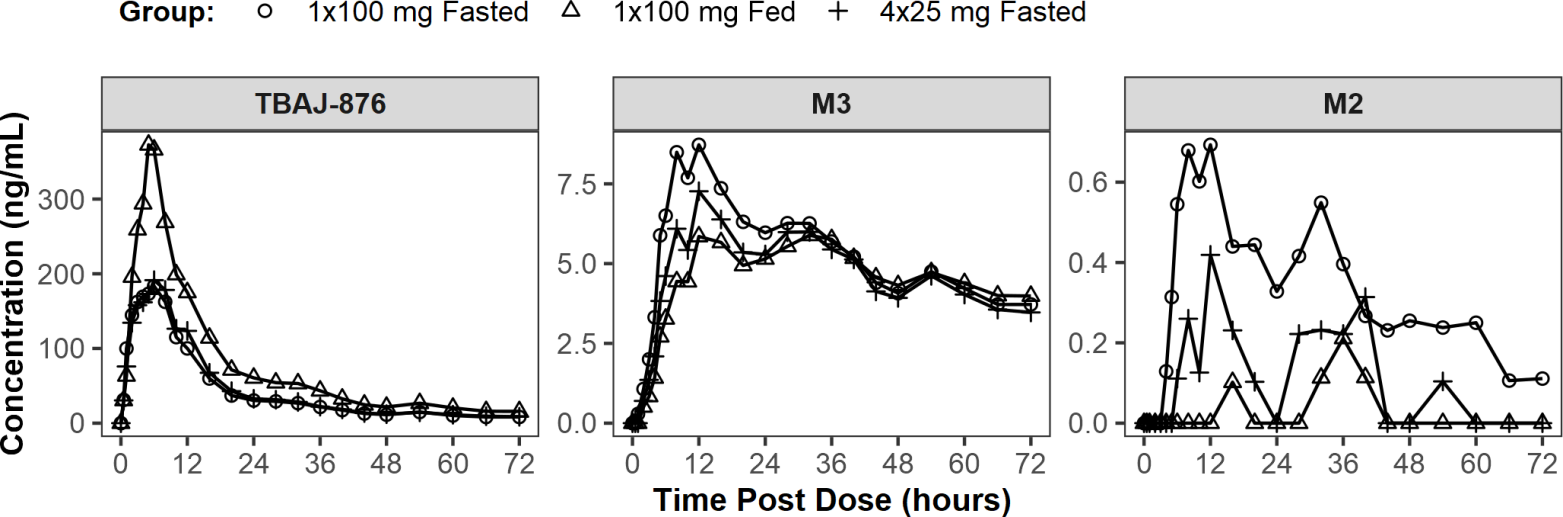
Mean plasma concentrations over time of TBAJ-876, M3, and M2 in the relative bioavailability part of CL-001, after single doses of 100 mg of TBAJ-876 administered as one 100-mg tablet fasted, one 100-mg tablet fed, and four 25-mg tablets fasted.

The mean plasma concentrations over time of TBAJ-876, M2, and M3 after escalating, single, fasted doses of 10 mg to 800 mg TBAJ-876 administered as an oral suspension are shown in Fig. 1; and so are profiles in fasted and fed states after 100-mg doses of the TBAJ-876 oral suspension. Profiles after administration of TBAJ-876 in the tablet formulation are summarized in Fig. 2. The noncompartmental PK parameters of TBAJ876 and M3, including food effects and rBA data, are summarized in Tables 2 and 3. M2 values are presented in Table S1.

**TABLE 2:** PK parameters of TBAJ-876: (CL-001 SAD, food effect, and relative BA)

Group	Fed or fasted		T_max_ (h)		C_max_ (ng/mL)		AUC_0-24h_ (h^*^ng/mL)		AUC_0-72h_ (h^*^ng/mL)	
		N	Median	Range	Mean	SD	Mean	SD	Mean	SD
10 mg suspension	Fasted	6	6.00	4.00–8.00	18.8	5.99	214	58.6	277	72.0
25 mg suspension	Fasted	6	5.99	2.99–8.00	54.3	20.4	588	213	765	276
50 mg suspension	Fasted	6	5.99	5.99–6.07	103	46.2	1180	530	1560	720
100 mg suspension	Fasted	9	6.00	5.00–6.12	215	56.5	2250	634	3000	863
1 × 100 mg tablet	Fasted	10	6.00	2.03–8.01	197	80.7	2310	974	4140^*a*^	2000^*a*^
4 × 25 mg tablet	Fasted	10	6.00	3.00–8.00	194	70.0	2460	823	4290	1310^*a*^
100 mg suspension	Fed	10	4.00	2.00–5.00	429	153	3640	1160	4810	1500
100 mg tablet	Fed	10	5.00	2.00–6.05	441	154	4040	968	7380^*a*^	1340^*a*^
200 mg suspension	Fasted	6	6.00	3.00–6.02	506	92.7	4990	929	6490	1220
400 mg suspension	Fasted	6	6.00	5.00–6.02	780	176	7190	1920	9780	2800
800 mg suspension	Fasted	6	5.99	4.01–6.00	1320	393	12400	3010	16400	3820

^*a*^ Data shown are AUC_last_, not AUC_0–72h._

**TABLE 3:** PK parameters of M3: CL-001 SAD, food effect, and relative BA

Group	Fed or fasted		T_max_ (h)		C_max_ (ng/mL)		AUC_0-24h_ (h^*^ng/mL)		AUC_0-72h_ (h^*^ng/mL)	
		N	Median	Range	Mean	SD	Mean	SD	Mean	SD
10 mg suspension	Fasted	NA^*a*^								
25 mg suspension	Fasted	5	12.0	7.99–12.1	2.85	0.806	42.1	14.6	115	35.6
50 mg suspension	Fasted	6	12.0	8.10–12.0	4.08	1.20	66.6	18.3	188	32.3
100 mg suspension	Fasted	9	12.0	6.12–16.0	10.5	2.02	162	29.1	429	83.1
1 × 100 mg tablet	Fasted	10	12.0	8.00–32.0	9.37	5.83	147	92.2	886^*b*^	475^*b*^
4 × 25 mg tablet	Fasted	10	12.0	12.0–32.0	7.47	2.76	117	44.6	816^*b*^	353^*b*^
100 mg suspension	Fed	10	12.0	5.00–32.0	5.04	1.75	85.5	30.6	250	86.4
100 mg tablet	Fed	10	16.0	5.00–40.0	6.49	1.49	98.3	21.9	931^*b*^	150^*b*^
200 mg suspension	Fasted	6	10.0	7.99–16.0	19.1	4.80	306	81.9	787	153
400 mg suspension	Fasted	6	12.0	6.05–12.0	31.0	8.04	511	111	1330	260
800 mg suspension	Fasted	6	11.0	8.01–12.0	49.1	21.2	821	368	2110	875

^*a*^ Data not available at this dose (due to values BLQ).

^*b*^ Data shown are AUC_last_, not AUC_0–72h._

Like other lipophilic anti-tuberculosis drugs such as bedaquiline, TBAJ-876 exhibited a long terminal half-life. The duration of PK follow-up varied across cohorts of the SAD part, from 1 week in Cohort 1 to 10 weeks in Cohort 6, as follow-up times were adjusted based on accumulating knowledge. Concentrations of TBAJ-876 were quantifiable (> 1 ng/mL) in all subjects who remained in the study through the scheduled last sample 10 weeks post-dose in Cohorts 4–6. Because concentrations in the observable elimination phase of the concentration–time profile did not decline steadily enough, individual values of half-life could not be calculated for all subjects across all cohorts. In Cohort 6 (400 mg), the half-life could be calculated for five of six subjects, and the mean value was 1,880 hours (standard deviation, 1,730 hours) (Table S2).

Varying duration of follow-up limits the comparability of AUC_last_ across cohorts, as well as of AUC_inf_; hence, AUC_last_ and AUC_inf_ should be interpreted with caution for the SAD (Tables S1 through S3). However, for all three cohorts of the rBA part, PK samples were collected for the same duration, 14 days, so AUC_last_ may be meaningfully compared across cohorts ( Tables S8 and S9).

Overall, after single doses of the suspension formulation in the fasted state, the mean TBAJ-876 exposure, as measured by C_max_ and AUC, increased proportionally with increases in the dose. The slope (β_1_) values for C_max_, AUC_0–24h_, and AUC_0–72h_ were 0.99, 0.94, and 0.94, respectively. The 90% CIs included 1.0 or nearly so for C_max_ and AUCs (Table S15).

The first quantifiable TBAJ-876 concentrations were observed at the first post-dose collection time (0.5 hours) across dose levels, indicating that TBAJ-876 was rapidly absorbed into systemic circulation. The peak mean TBAJ-876 concentrations were generally observed between 5 and 6 hours post-dose, or 1 hour earlier under fed conditions, and declined thereafter in a multiphasic manner across dose levels, with minor secondary peaks on the second and third days after dosing, particularly at the higher doses (Fig. 1).

Mean TBAJ-876 C_max_ values ranged from 18.8 ng/mL after 10 mg to 1,320 ng/mL after 800 mg. The partial AUCs ranged from 214 h*ng/mL to 12,400 h*ng/mL for AUC_0–24h_ and from 277 h*ng/mL to 16,400 h*ng/mL for AUC_0–72h_.

Overall, mean M3 exposure, as measured by C_max_ and AUCs, increased in a slightly less than proportional manner with an increase in the TBAJ-876 dose as the oral suspension under fasting conditions from 25 mg to 800 mg. The slope (β_1_) values for C_max_, AUC_0–24h_, and AUC_0–72h_ were 0.85, 0.88, and 0.86, respectively, and the 90% CIs did not include 1.0. Only two quantifiable M3 concentrations were reported for the 10-mg dose cohort, which was therefore excluded from PK analysis.

The first quantifiable M3 concentrations were observed between 1 and 3 hours post-dose. Peak mean concentrations were observed between 8 and 12 hours post-dose for M3. M3 concentrations declined in a multiphasic manner, with secondary peaks on the second and third days after dosing (Fig. 1).

Mean M3 C_max_ values ranged from 2.85 ng/mL after 25 mg to 49.1 ng/mL after 800 mg. The partial AUCs ranged from 42.1 h*ng/mL to 821 h*ng/mL for AUC_0–24h_ and from 115 h*ng/mL to 2110 h*ng/mL for AUC_0–72h_.

For the 25-mg and 100-mg tablet formulation under fasted conditions, peak mean TBAJ-876 concentrations were similar between 1 × 100 mg and 4 × 25 mg and occurred at 6 ours post-dose for both groups (Fig. 2). Peak mean concentrations of M3 were slightly higher after 1 × 100 mg compared to 4 × 25 mg tablets and occurred at 12 hours post-dose for both groups. After approximately 36 hours, mean M3 concentrations were similar across groups (Fig. 2). Geometric means of exposure metrics for TBAJ-876 and M3 differed by less than 20% between 4 × 25 mg tablet and 1 × 100 mg tablet administrations under fasting conditions (Supplemental Material, Tables S8 and 9).

Exposures of TBAJ-876 and M3 arising from administration of 100 mg of the oral suspension and tablet formulations were similar, albeit with a 29% higher C_max_ for M3 with the tablet versus the suspension under fasting conditions (Tables 2 and 3; Table S10).

Geometric mean TBAJ-876 maximum and total exposure (C_max_, AUC_0–24h_, and AUC_0–72h_) increased by 96%, 60%, and 59%, respectively, after administration of TBAJ-876 with food relative to the administration under fasted conditions with the oral suspension (Table S4). For the tablet, geometric mean values of C_max_, AUC_0–24h_, and AUC_last_, were 132%, 85%, and 94% higher, respectively, under fed conditions compared to fasted conditions (Table S6).

In contrast to TBAJ-876, the M3 exposures were mostly lower in the fed state than in the fasting state at doses of 100 mg. Geometric mean M3 maximum and total exposure (C_max_, AUC_0–24h_, and AUC_0–72h_) decreased by 54%, 50%, and 44%, respectively, after administration of TBAJ-876 with food relative to administration under fasted conditions with the oral suspension (Table S5). For the tablet, geometric mean values of C_max_, AUC_0–24h_, and AUC_last_ were 19% and 21% lower and 27% higher, respectively, under fed conditions compared to fasted conditions (Table S7). For all three metrics, the 90% confidence intervals (CIs) included no difference.

#### CL-001: multiple-dose PK

**Fig 3:**
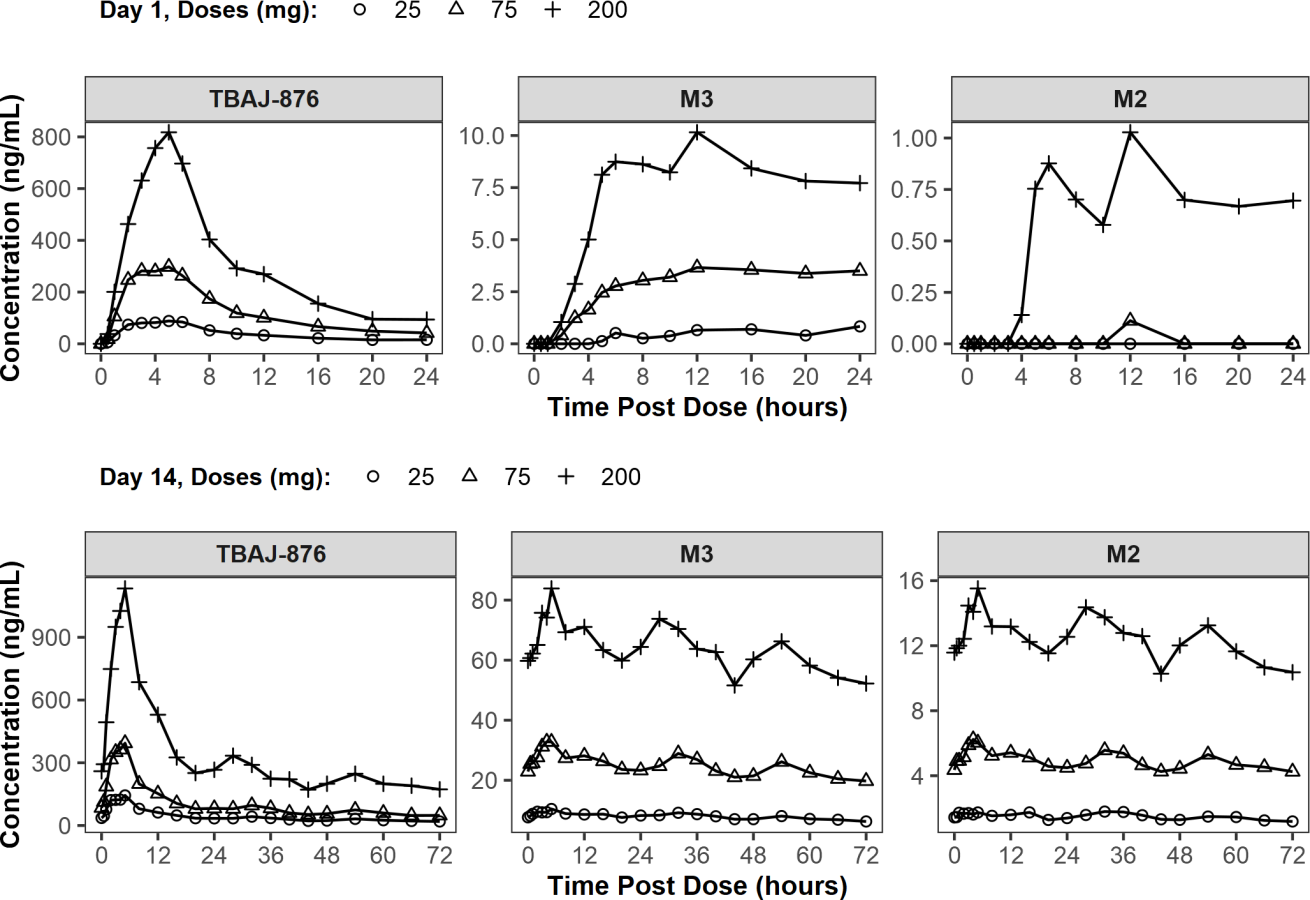
Mean plasma concentrations over time of TBAJ-876, M3, and M2 in the MAD part of CL-001. Top: Day 1. Bottom: Day 14. TBAJ-876 administered daily as an oral suspension under fed conditions.

All MAD doses were administered as an oral suspension under fed conditions. Concentration–time data for TBAJ-876, M2, and M3 are compared across doses for Day 1 in Fig. 3. Concentration–time data are compared across time for each dose cohort in, Figures S1 through S3. Day 1 and Day 14 PK parameters of TBAJ-876 and M3 are summarized in Tables 4 and 5.

**TABLE 4:** PK parameters of TBAJ-876: CL-001 MAD Day 1 and Day 14

Group	Fed or fasted		T_max_ (h)		C_max_ (ng/mL)		AUC_0-24h_ (h*ng/mL)		C_24_ (ng/mL)	
		N	Median	Range	Mean	SD	Mean	SD	Mean	SD
Day 1										
25 mg suspension	Fed	9	3.01	2.00–6.02	95.9	30.8	938	231	12.3	3.65
75 mg suspension	Fed	9	4.99	1.99–5.99	322	106	3020	691	36.5	7.78
200 mg suspension	Fed	9	4.99	3.00–6.00	873	343	7270	2190	75.0	20.6
Day 14										
25 mg suspension	Fed	9	5.00	2.00–5.01	148	47.0	1670	480	34.1	8.27
75 mg suspension	Fed	9	5.00	2.07–5.01	401	122	4240	1110	81.9	25.8
200 mg suspension	Fed	9	5.00	3.01–5.02	1160	502	12900	5780	266	93.6

**TABLE 5:** PK parameters of M3: CL-001 MAD Day 1 and Day 14

Group	Fed or fasted		T_max_ (h)		C_max_ (ng/mL)		AUC_0-24h_ (h^*^ng/mL)		C_24_ (ng/mL)	
		N	Median	Range	Mean	SD	Mean	SD	Mean	SD
Day 1										
25 mg suspension	Fed	6	20.0	6.00–23.9	1.33	0.314	19.5	NC^*a*^	1.02	NC
75 mg suspension	Fed	9	20.0	6.00–24.0	3.96	1.72	81.9	37.2	3.75	0.975
200 mg suspension	Fed	9	12.0	5.00–23.9	11.0	5.48	191	81.0	7.90	2.90
Day 14										
25 mg suspension	Fed	9	5.00	2.00–16.0	10.6	3.96	209	77.2	8.25	3.04
75 mg suspension	Fed	9	5.00	0.499–12.0	35.2	7.70	649	133	23.3	5.35
200 mg suspension	Fed	9	5.00	3.01–8.00	85.8	27.2	1620	459	64.4	19.1

^*a*^ NC, not calculated.

On both Day 1 and Day 14, maximum and total exposure (C_max_ and AUC_0–24h_) of TBAJ-876 and M3 increased in a proportional manner with an increase in the TBAJ-876 dose over the 25-mg to 200-mg dose range. The slope (β_1_) estimates ranged from 0.96 to 1.05, and all 90% CIs included 1 (Tables S16 through S20).

For TBAJ-876, mean C_max_ values ranged from 95.9 ng/mL to 873 ng/mL on Day 1 and from 148 ng/mL to 1,160 ng/mL on Day 14; and mean AUC_0-24h_ values ranged from 938 h*ng/mL to 7,270 h*ng/mL on Day 1 and from 1,670 h*ng/mL to 12,900 h*ng/mL on Day 14 (Table 4). Median values of T_max_ were similar across doses and days, ranging from 3 hours to 5 hours post-dose.

For M3, mean C_max_ values ranged from 1.33 ng/mL to 11.0 ng/mL on Day 1 and from 10.6 ng/mL to 85.8 ng/mL on Day 14; and mean AUC_0–24h_ values ranged from 19.5 h*ng/mL to 191 h*ng/mL on Day 1 and from 209 h*ng/mL to 1,620 h*ng/mL on Day 14 (Table 5). Median values of T_max_ were longer on Day 1 compared to Day 14; median T_max_ values ranged from 12 hours to 20 hours post-dose on Day 1 and were 5 hours on Day 14.

Based on mean values of AUC_0–24h_ after 14 days of dosing 200 mg, ratios of M2 and M3 to the parent were 0.0240 and 0.126, respectively, (Tables S12 through S14).

Overall, mean concentration–time profiles were higher after once-daily dosing over 14 days (Day 14) compared to single dosing (Day 1) for all three cohorts and analytes (Figures S1 and S3). Accumulation was assessed by examining daily trough concentrations and ratios of exposure metrics on Day 14 versus Day 1. As per results from Tukey’s multiple comparison test (Table S11), the hypothesis of no further increase in TBAJ-876 trough concentrations could not be rejected (*P* > 0.05), starting at Days 9, 6, and 6 in Cohorts 1, 2, and 3, respectively. Note that failure to reject the hypothesis of no further increase in trough concentrations does not prove that there were no further increases. There is evidence that the half-life of TBAJ-876 is longer than 14 days, perhaps substantially longer, in which case steady state would not be reached after only 14 days of dosing.

For TBAJ-876, accumulation ratios for C_max_ ranged from 1.27 to 1.60; for AUC_0–24h_ from 1.41 to 1.78; and for C_24_ from 2.28 to 3.61 (Table S12). For M3, accumulation ratios for C_max_ ranged from 8.69 to 9.65; for AUC_0–24h_ from 9.16 to 11.0; and for C_24_ from 7.54 to 8.41 (Table S14).

#### CL-002: DDI study

**Fig 4:**
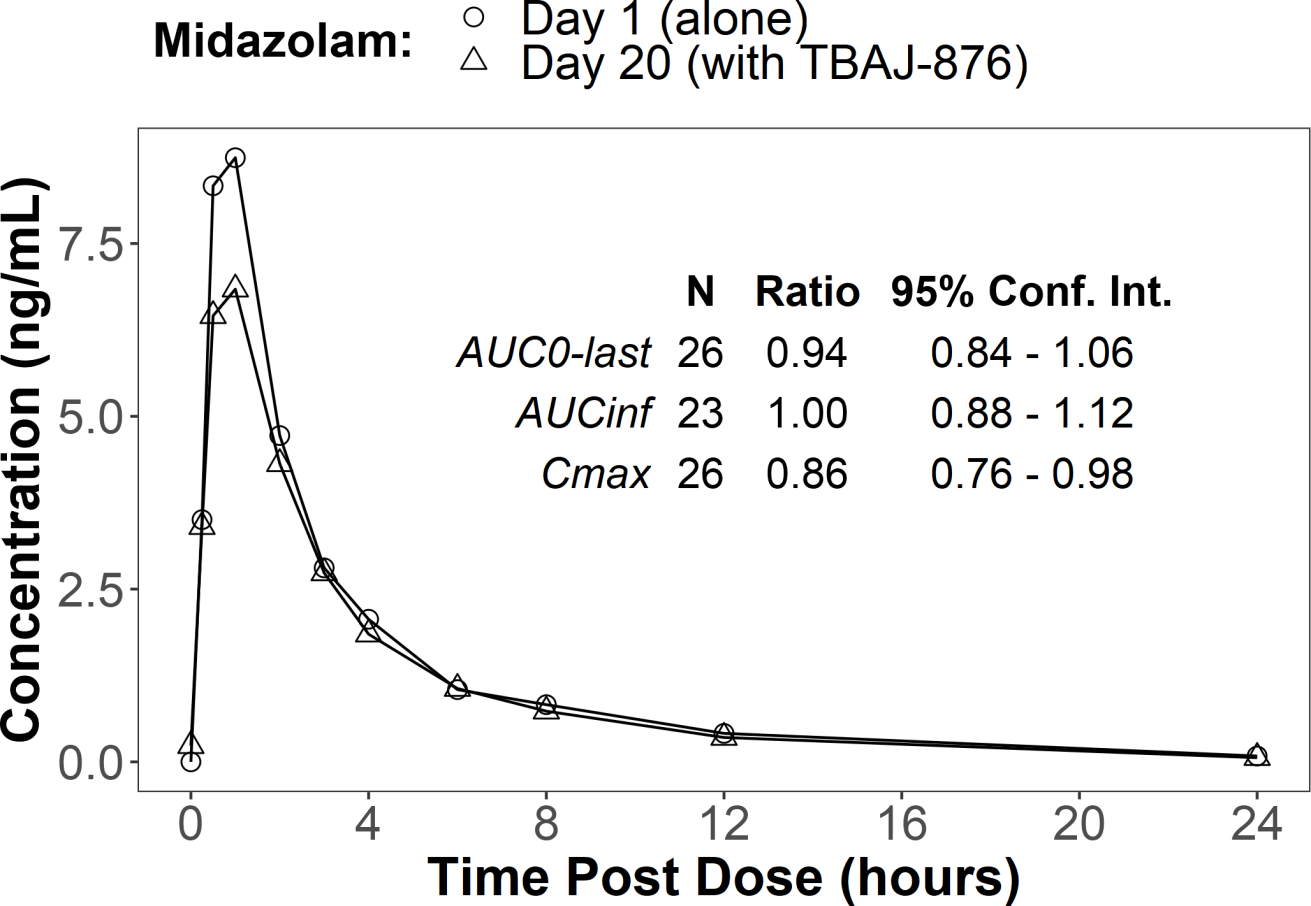
DDI study, midazolam alone (Day 1) and with TBAJ-876 (Day 20). Mean plasma concentrations over time and geometric mean ratios with 90% confidence intervals (CIs).

**Fig 5:**
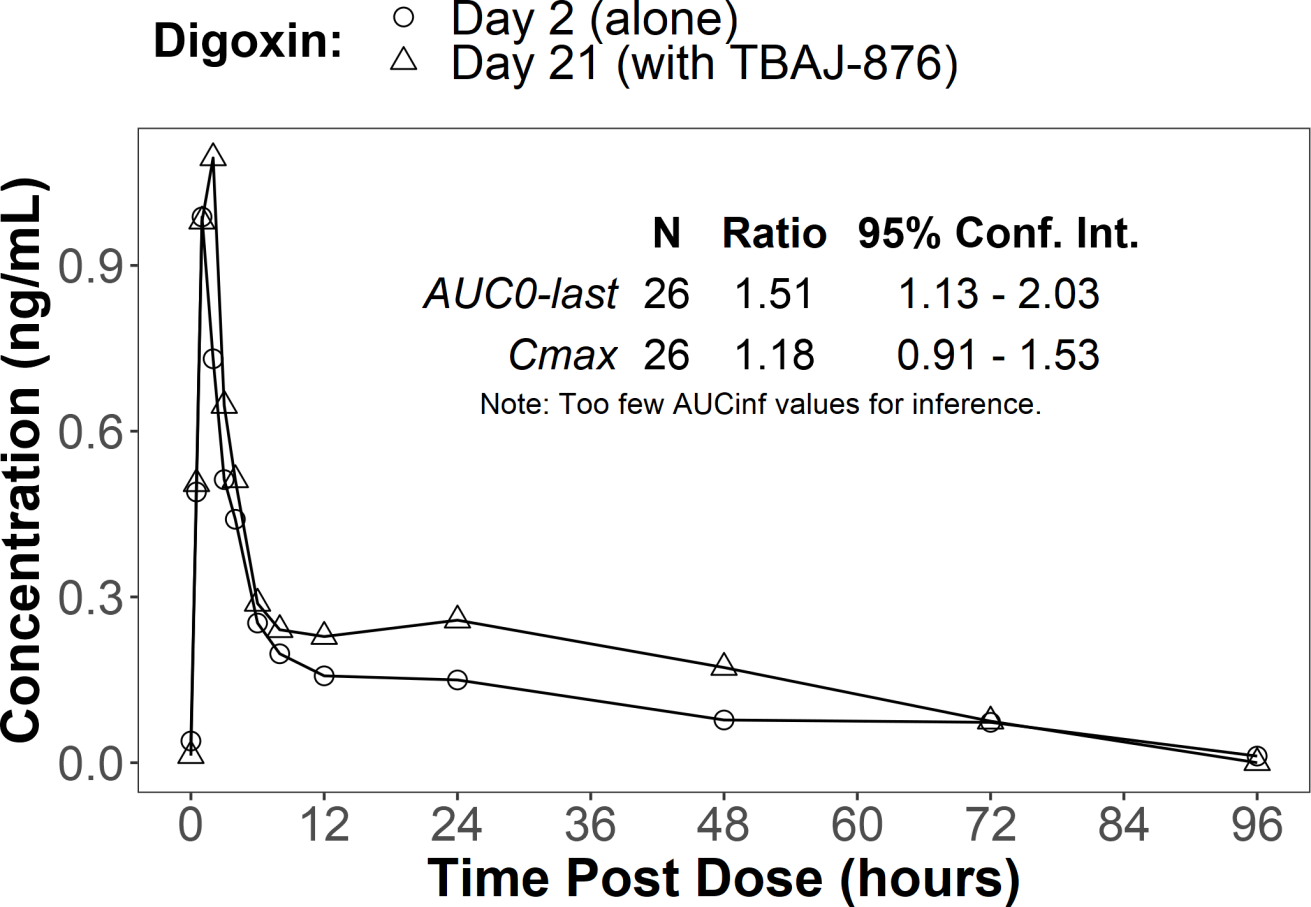
DDI study, digoxin alone (Day 2) and with TBAJ-876 (Day 21). Mean plasma concentrations over time and geometric mean ratios with 90% confidence intervals (CIs).

CL-002 assessed the induction potential of TBAJ-876 on the sensitive CYP3A4 substrate midazolam and inhibition and induction potential of TBAJ-876 on the sensitive P-gp substrate digoxin. With co-administration of TBAJ-876 after 14 days of TBAJ-876 dosing, the AUC_0-inf_ of midazolam was unchanged, and the C_max_ was reduced by 14% (Fig. 4); the AUC_0-last_ of digoxin was increased by 51%, and the C_max_ was increased by 18% (Fig. 5). After 12 days of TBAJ-876 administration using a regimen designed to reach and sustain anticipated clinical exposures, the mean (SD) of TBAJ-876 AUC_0–24h_, C_max_, and C_trough_ were 14,620 (5,531) ng.h/mL, 1,224 (513) ng/mL, and 293 (110 ng/mL), respectively (Table S21).

### Safety

#### CL-001

TBAJ-876 was generally safe and well-tolerated. There were no fatal or serious treatment emergent adverse events (TEAEs) (Table S22). Very few of the TEAEs were Grade 2 or 3 (Table 6), and all were resolved. In both the SAD and MAD cohorts, the TEAE profile was generally similar in the active and placebo groups (Table S22).

**TABLE 6:** Grade 2 or 3 treatment emergent adverse events, CL-001 All Three Parts

Adverse event	TBAJ-876 (*n* = 112)	Placebo (*n* = 25)
Investigations		
Increased lipase	2	2
Increased amylase	0	1
Increased ALT	2	0
Proteinuria	1	0
Musculoskeletal		
Rhabdomyolysis	1	0
Pain in extremity	1	1
Muscle spasms	1	0
Skin		
Dermatitis contact	0	1
Other		
Pyrexia	0	1

There were no reported instances of clinically significant QT prolongation, nor were differences noted in the change from baseline in QTc at 6 hours post-dose between subjects on placebo or on any dose of TBAJ-876 (Table 7). There was also no evidence of drug-induced myocardial, hepatic, or pancreatic toxicity (Table S22). One participant who received a single dose of 400 mg TBAJ-876 experienced a brief episode of non-sustained ventricular tachycardia on Day 2. This participant was later withdrawn from the trial due to a urine screen test being positive for cocaine.

**TABLE 7:** Mean QTcF intervals and change from baseline at 6 hours post-dose in CL-001 cohorts

Group		QTcF		ΔQTcF	
	n	Mean	SD	Mean	SD
SAD and rBA, Day 1					
Placebo	13	407.6	17.18	0.8	9.71
10 mg	6	411.2	16.07	3.7	17.04
25 mg	6	412.8	18.05	−3.0	11.08
50 mg	6	409.8	16.40	1.2	13.82
100 mg fasted	9	409.1	10.90	−0.8	6.87
1 × 100 mg tab. fasted	10	405.0	14.02	0.3	5.72
4 × 25 mg tab. fasted	10	396.4	12.82	−2.7	5.74
100 mg susp. fed	10	414.0	17.51	5.9	9.61
100 mg tab. fed	10	392.3	12.98	−1.6	6.28
200 mg	6	418.5	18.53	−0.8	8.70
400 mg	6	403.2	13.00	5.8	7.31
800 mg	6	416.0	10.99	2.3	5.28
MAD, Day 14					
Placebo	12	414.3	17.93	0.1	18.78
25 mg	9	405.1	14.04	7.3	6.28
75 mg	9	402.7	23.41	5.6	15.39
200 mg	9	404.1	17.29	−2.6	9.58

In the SAD part, two participants (in the 25-mg and 400-mg cohorts, respectively) experienced marked elevation in serum CPK, reported as rhabdomyolysis, on Day 10 and 70, respectively. In both cases, participants reported mild muscular pain after strenuous physical exertion. There were no signs of impaired renal function, and the CPK elevations resolved within 1–3 weeks. In the MAD portion, a few participants reported myalgia or muscle spasms (Table S22) that were not associated with changes in muscle enzymes and resolved within 1–2 days.

#### CL-002

Overall, TBAJ-876 administered alone or in combination with digoxin and midazolam was generally safe and well-tolerated (Table S23). Very few adverse events were Grade 2 or 3 (Table 8). As in CL-001, there were no episodes of clinically significant prolongation of the QTc interval.

**TABLE 8:** Grade 2 or 3 treatment emergent adverse events, CL-002

Adverse event	TBAJ-876 (*n* = 28)
Neurologic	
Headache	3
Infections	
Pneumonia	1
Respiratory	
Cough	1
Musculoskeletal	
Flank pain	1
Other	
Pyrexia	1
Chest discomfort	1

## DISCUSSION

TBAJ-876’s safety and tolerability, PK profile, and robust *in vitro* and *in vivo* bactericidal potency suggest its potential superiority to bedaquiline and support further development. Preclinical data suggest a reduced risk for QT prolongation compared to bedaquiline. A subsequent phase 2 trial, NC-009 (NCT06058299), is designed to evaluate three dose levels of TBAJ-876–25, 50, and 100 mg QD—for 8 weeks in combination with pretomanid (Pa) and linezolid (L), compared to 8 weeks of isoniazid, rifampicin, pyrazinamide, and ethambutol (HRZE), in adult participants with newly diagnosed, smear-positive, pulmonary DS-TB. Also compared will be a fifth arm of bedaquiline plus Pa and L, the BPaL combination that is part of the WHO’s recommended treatment strategy for rifampicin-resistant TB (2). This will provide a head-to-head comparison of TBAJ-876 and bedaquiline in participants with DS-TB.

In the two phase 1 studies reported here, the safety and tolerability profile of TBAJ-876 was unremarkable and not distinguishable from that of subjects assigned placebo. No specific findings attributable to TBAJ-876 that reflected toxicologic findings in preclinical studies were observed, where target organs included skeletal and cardiac muscle, pancreas, gastrointestinal (GI) tract, liver, and bone marrow. All potential risks will continue to be monitored in future studies to establish further the safety profile of TBAJ-876.

In terms of PK, TBAJ-876 and bedaquiline are qualitatively similar, exhibiting dose-proportional, multicompartmental behavior with long terminal half-lives (9). The terminal half-life of bedaquiline is approximately 5.5 months (10). Estimation of the TBAJ-876’s half-life in CL-001 was problematic, with 1,880 hours, or about 2.5 months, being our favored estimate. If this is indeed near the truth, then 10 weeks of follow-up, the maximum duration in the SAD, may not yield a reliable estimate (11), but it was unrealistic to follow subjects longer in a healthy volunteer study. Further exploration of the terminal half-life will be undertaken in later studies. Such long terminal half-lives may be beneficial, if continued exposure helps eliminate persisting bacteria after treatment formally ends, or problematic, if continued low-level exposure promotes the selection of resistance.

As with bedaquiline’s active M2 metabolite (9), the active M3 metabolite of TBAJ-876 appears at higher exposures relative to the parent in preclinical species compared to humans. In humans, the exposure of M2 is approximately 22%–28% that of bedaquiline after 2 weeks of dosing (12). The corresponding figure for TBAJ-876 is approximately 13% (Tables 4 and 5), but the greater potency of M3 (4) may yield a relatively greater contribution to efficacy than from M2.

In conclusion, these two phase 1 studies of TBAJ-876 provide pharmacokinetic and safety data that support its advancement to phase 2, wherein its promise as a second-generation diarylquinoline, on the basis of its superior potency, can be explored.

## MATERIALS AND METHODS

### Study design

The protocol, informed consent form, and other study documents for CL-001 and CL-002 were reviewed and approved by IntegReview Institutional Review Board, Austin, TX, and by Advarra, Columbia, MD, United States, respectively. CL-001 was conducted at a single site in San Antonio, TX, United States, and CL-002 at a single site in Fair Lawn, NJ, United States, under the sponsorship of TB Alliance.

#### CL-001, dose selection

The starting single dose of 10 mg was selected because based on body surface area extrapolation, it was one-tenth the human equivalent dose (HED) for the 13-week no adverse effect level (NOAEL) in rats and one-eighth of the HED for the 13-week NOAEL in dogs. The high dose in the SAD was tentatively set at 400 mg in the sixth cohort, with the allowance for all dose levels after the first to be adjusted based on safety, tolerability, and pharmacokinetics. As it turned out, the first six cohorts proceeded as planned. Dose selection for the MAD was guided by the predicted sum of the AUCs of TBAJ-876 and M3 (SAUC) because of the greater formation in M3 in nonclinical species. Mean SAUC_0–24h_ at NOAEL doses in the dog and rat were 27.2 µg.h/mL and 13.6 µg.h/mL, respectively. Mean SAUC_0–24h_ in the dog at the dose where cardiotoxicity was observed was 128 µg.h/mL. When a high dose for the MAD of 100 mg–200 mg was projected, the seventh SAD cohort at 800 mg was added to assure prior coverage of the anticipated high-dose Cmax in the MAD. The maximum mean SAUC_0–24h_ at the 200 mg dose in the MAD was 14.5 µg.h/mL.

#### CL-001, SAD, and Food-Effect Part

Part 1 of CL-001 was a randomized, placebo-controlled, combined SAD investigation with seven fasting cohorts and a food-effect cohort. Doses of 10 mg, 25 mg, 50 mg, 100 mg, 200 mg, 400 mg, and 800 mg of TBAJ-876 in a suspension formulation or placebo were administered under blinded, fasting conditions in Cohorts 1–7. There were seven–eight subjects per cohort (six received active drug and two received placebo [except in Cohort 4 where only one received placebo]). Based on observed PK and safety, 100 mg was selected to assess the effect of food. Three additional subjects received 100 mg TBAJ-876 fasted and ten additional subjects received 100 mg TBAJ-876 fed, in unblinded conditions. Subjects were housed in the clinic from at least 48 hours prior until 7 days after dosing. Different cohorts were followed-up for 3–10 weeks.

For all cohorts, physical examination (including heart murmurs), vital signs, ECGs, extensive cardiac monitoring including telemetry and Holter monitoring, AEs, hematology, serum chemistry, coagulation, and urinalysis were used to assess the safety and tolerability. Blood and urine samples were collected for clinical laboratory evaluations. Female subjects had blood collected for serum pregnancy testing. Females claiming postmenopausal status had blood collected to measure follicle stimulating hormone levels. Given findings in the nonclinical safety studies of minimal and mild cardiovascular, liver, and skeletal muscle injury, extensive monitoring was conducted with emphasis on cardiovascular monitoring as well as laboratory evaluation of potential hepatocellular and skeletal muscle injury. This occurred at both protocol-defined time points and whenever deemed necessary by the study investigator.

At the end of Part 1, PK and safety data were sent to the Institutional Review Board and the FDA for review and approval prior to proceeding to Part 2.

#### CL-001, MAD Part

Part 2 of CL-001 was a randomized, blinded, placebo-controlled, MAD investigation with three cohorts. Cohort 1 (25 mg) included 12 subjects (nine received the active drug and three received placebo), Cohort 2 (75 mg) included 13 subjects (nine received the active drug and four received placebo), and Cohort 3 (200 mg) included 14 subjects (nine received the active drug and five received placebo). TBAJ-876 was administered in a suspension formulation under blinded, fed conditions once daily for 14 days. After the last dose, subjects remained in the clinic for 7 additional days and then returned every 3 weeks (Days 42, 63, 105, and 126) for additional PK and safety assessments.

#### CL-001, relative BA Part

Part 3 of CL-001 assessed the PK of two strengths, 100 mg and 25 mg, of a tablet formulation of TBAJ-876. The study consisted of three parallel, open-label groups of 10 subjects each. Group 1 received 1 × 100 mg tablet fasted, Group 2 received 1 × 100 mg tablet fed, and Group 3 received 4 × 25 mg tablets fasted. Evaluations included the effect of food on the 100-mg tablet, the relative BA of the 100-mg tablet compared to the suspension under fed and fasted conditions (by comparison with data from Part 1), and the relative BA of the 25-mg strength tablet compared to the 100 mg strength. After the last dose, subjects remained in the clinic for 7 additional days.

#### CL-002, DDI Study

The DDI study evaluated the potential effects of TBAJ-876 on CYP3A4 and P-gp. Midazolam was the probe substrate for CYP3A4 and digoxin for P-gp. Twenty-eight subjects were randomized, and 26 subjects completed the study. The subjects were treated with the following regimen:

- On Days 1 and 20, subjects received 2 mg midazolam with 24 hours of PK sampling after each dose.
- On Days 2 and 21, subjects received 0.25 mg digoxin with 96 hours of PK sampling after each dose.
- On Days 6–13 and 14–19, subjects received 200 and 165 mg TBAJ-876 in the fed state, respectively; on Days 20–2, they received 200 mg TBAJ-876 in the fasted state; and on Days 22–24, they received 150 mg TBAJ-876 in the fed state. This regimen was designed to reach and sustain anticipated clinical exposures, while allowing dosing in the fasting state on the days of midazolam and digoxin co-administration. Twenty-four hours of PK sampling for TBAJ-876 was conducted on Day 17.
- On Day 25, subjects were discharged from the clinic following completion of all procedures.
- On Day 32, subjects received a follow-up phone call to check for AEs and any concomitant medications.

#### Exclusion criteria

For CL-001, the health status was assessed by history and physical examination, ECG, serum/urine clinical chemistry, hematology, and serology tests. Subjects were excluded if they had a body mass index (BMI) outside the range of 18.5 to 32 kg/m^2^ or weight <50 kg; a history of clinically significant cardiovascular or musculoskeletal disease; a history of any clinically significant laboratory abnormalities, including elevated ALT, AST, total bilirubin, creatinine phosphokinase, pancreatic amylase or lipase, or positive hepatitis B, hepatitis C or HIV; alcohol and/or substance abuse within the past 2 years; recent tobacco use; exposure to prescription medications within 14 days, over-the-counter medications, herbal medications, or vitamin supplements except acetaminophen within 7 days; a finding of clinically significant ECG abnormalities or a finding of abnormal pulse or blood pressure on repeated testing at screening; a QTcF (QT interval corrected for heart rate by Fridericia’s formula) interval >450 msec for males or >470 msec for females at screening, Day −1, or Day 1 (pre-dose), or history of prolonged QT syndrome; family history of prolonged QT syndrome or unexplained sudden death. Subjects were also excluded if they had used any significant inhibitors of CYP enzymes and/or significant inhibitors or substrates of P-gp and/or organic anion transporting polypeptides within 14 days and any inducers of CYP enzymes and/or P-gp, including St. John’s Wort, within 30 days prior to the first dose of TBAJ-876.

For CL-002, exclusion criteria were similar to CL-001. In addition, subjects who were positive for SARS-CoV-2 were excluded within 6 days prior to Day 1.

### Assessments

#### Blood sampling

##### CL-001

In all parts and cohorts, post-dose sampling for PK occurred at 0.5, 1, 2, 3, 4, 5, 6, 8, 10, 12, 16, 20, and 24 hours. In the SAD and rBA parts, additional samples were then collected at 28, 32, 36, 40,48, 54, 60, 66, 72, 80, 88, 96, 120, 144, and 168 hours post-dose. Additional sampling of varying durations through 70 days was then conducted in some single-dose cohorts. In the MAD part, pre-dose trough concentrations were collected daily through Day 13, and then on Day 14, a profile was collected similarly to Day 1 through 24 hours post-dose. Complete sampling schedules are provided in the Supplemental Material (Table S24).

##### CL-002

In the DDI study, blood samples for safety were drawn pre-dose and on Days 2, 3, 7, 20, 21, 22, and 25. PK assessments were drawn for midazolam on Days 1 and 20, pre-dose, and at 0.25, 0.5, 1, 2, 3, 4, 6, 8, 12, and 24 hours; for TBAJ-876 on Day 17, pre-dose, and at 0.5, 3, 4, 5, 6, 7, 8, 12, 16, 20, and 24 hours; and, for digoxin, on Day 2, pre-dose, and at 0.5, 1, 2, 3, 4, 6, 8, 12, 24 (Day 3), 48 (Day 4), 72 (Day 5), and 96 (Day 6) hours and on Day 21, pre-dose, and at 0.5, 1, 2, 3, 4, 6, 8, 12, 24 (Day 22), 48 (Day 23), 72 (Day 24), and 96 (Day 25) hours.

### Bioanalytical procedures

Blood was collected into prechilled 4-mL evacuated tubes containing K_3_-EDTA, gently and immediately placed on wet ice, and then processed to plasma within 60 minutes. Samples were centrifuged at 1,500 g at approximately 4°C (±10°C) for 10 minutes. After centrifugation, two aliquots of plasma (the first containing at least 0.5 mL and the second containing the remainder of the plasma) were removed and placed in appropriately labeled 1-mL polypropylene vials. The aliquots were immediately placed on dry ice. Within 90 minutes of collection, aliquots were stored in a freezer set at −80°C (±10°C) until transferred on dry ice to Alliance Pharma, Inc. (now Resolian), for bioanalytical analysis.

TBAJ-876, M2, and M3 plasma concentrations were determined with a validated high-performance liquid chromatography–tandem mass spectrometry method within the same sample. The linear range of the method was 1–1,000 ng/mL for TBAJ-876, M2, and M3. Observations below the lower limit of quantification (LLOQ), i.e., 1 ng/mL, were considered below the quantification limit (BQL). A minimum of six quality control samples were included in each analysis, and incurred sample re-analysis (ISR) was within the 20% assay variability criterion for greater than 90% of samples for TBAJ-876, M2, and M3, thereby adhering to the condition of two-thirds of the repeats needing to be within the variability defined by the ICH-M10 guidance.

Standard noncompartmental PK parameters were calculated for TBAJ-876 and its M2 and M3 metabolites using Phoenix WinNonlin (Version 8.3.4.295, Certara, L.P.) (CL-001) and SAS (Version 9.4, SAS Institute, Inc.) (CL-002). In CL-001, for calculating the apparent elimination rate constant λ_z_ from the terminal log-linear segment of the plasma concentration–time curve, the range of data used was determined by visual inspection of a semi-logarithmic plot of concentration vs time, subject to the following criteria: a) at least three quantifiable concentrations were used in the regression; b) C_max_ or data prior to C_max_ were not included in the regression; c) the adjusted regression coefficient (R^2^ adj) was ≥0.80. If these acceptance criteria were not met, parameters calculated using λ_z_ (e.g., t_1/2_, AUC_inf_, CL/F, CL_ss_/F, and V_z_/F) were reported as ND (not determinable).

### Statistical analysis

Statistical assessments were performed on TBAJ-876, M2, and M3 using SAS (Version 9.4, SAS Institute, Inc.).

#### Dose proportionality

In CL-001, the PK parameters for TBAJ-876, M2, and M3 C_max_, AUC_last_, AUC_0-24h_, AUC_0_-_72h_, and AUC_inf_ (SAD), C_max,_ and AUC_0-24h_ (MAD, Days 1 and 14) were compared across doses to assess the dose proportionality. Statistical analyses were performed using a power model (13) of the following general form:


$$ln(PK)=ln(\beta_{0})+\beta_{1}\cdot ln(Dose)+\epsilon$$

where

PK is the pharmacokinetic parameter tested (e.g., C_max_ or AUC)

ln(β_0_) is the y-intercept, β_1_ is the slope, and ϵ is an error term

The estimates of β_1_ with the 90% CIs were reported along with the associated *P*-value. A β_1_ value of approximately 1 indicates linearity. Dose-proportionality plots were also created.

#### Food effects

In the SAD and rBA parts of CL-001, the effect of food was assessed by statistical comparison of the PK parameters of TBAJ-876, M2, and M3 using an analysis of variance (ANOVA) model for a parallel group design on the ln-transformed data with treatment as a fixed effect. Conclusions regarding the results of the statistical analysis of PK parameters across treatments were based on the ratio of the geometric means expressed as a percent and the 90% CI about the ratio. In the SAD part, the comparison of interest was TBAJ-876, 100 mg oral suspension fed vs TBAJ-876, 100 mg oral suspension fasted. In the rBA part, the comparison of interest was TBAJ-876, 1 × 100 mg tablet fed vs TBAJ-876, 1 × 100 mg tablet fasted.

#### Relative BA

The relative BA for the two strengths of TBAJ-876 tablets administered under fasted conditions was assessed for TBAJ-876, M2, and M3 C_max_ and AUCs using an ANOVA model for a parallel study design with the strength of the tablet administered under fasting conditions as the fixed effect. The comparison of interest was TBAJ-876, 4 × 25 mg tablets vs TBAJ-876, 1 × 100 mg tablet. The tablet formulation and the oral suspension were compared descriptively.

#### DDI study

To compare PK parameters of the interaction products with TBAJ-876 versus alone, ANOVA was performed by the SAS Mixed Linear Models procedure. The model included subject as a random effect and treatment (with TBAJ-876 versus alone) as a fixed effect. Following log transformation, geometric least-squares mean values and 95% CIs were tabulated for each PK parameter. Geometric mean ratios and 95% CIs were calculated for C_max_, AUC_inf_, and AUC_last_ of interaction products, with TBAJ-876 versus alone.

## SUPPLEMENTAL MATERIAL

Supplemental material aac.00613-24-s0001.pdf Tables S1 to S24; Figures S1 to S3.

ASM does not own the copyrights to Supplemental Material that may be linked to, or accessed through, an article. The authors have granted ASM a non-exclusive, world-wide license to publish the Supplemental Material files. Please contact the corresponding author directly for reuse.
