# Supplementary material for: Pharmacokinetics and safety of TBAJ-876, a novel antimycobacterial diarylquinoline, in healthy subjects
Source: Antimicrob Agents Chemother. 2024 Aug 28;68(10):e00613-24. doi: 10.1128/aac.00613-24 (PMC11460996; doi:10.1128/aac.00613-24)
Supplement: Supplemental material — Tables S1 to S24; Figures S1 to S3. [file aac.00613-24-s0001.pdf]

SUPPLEMENTAL MATERIAL
for

Pharmacokinetics and safety of TBAJ-876, a novel antimycobacterial diarylquinoline, in healthy subjects

Antonio Lombardi[a,#], Fran Pappas[a], Jerry Nedelman[a], Dean Hickman[a], Sarah Jaw-Tsai[b], Morounfolu Olugbosi[a], Paul Bruinenberg[c], Maria Beumont[a], Eugene Sun[a]

[a]Global Alliance for TB Drug Development, New York, New York, USA

[b]Sarah Jaw-Tsai Consulting Services, San Francisco, California, USA

[c] Vast Therapeutics

Contents

SUPPLEMENTAL MATERIAL 1

Supplemental Tables 4

Supplemental Table S1. CL-001 SAD (Part 1): Plasma Pharmacokinetic Parameters of M2 4

Supplemental Table S2. CL-001 SAD (Part 1): Plasma Pharmacokinetic Parameters of TBAJ-876 5

Supplemental Table S3. CL-001 SAD (Part 1): Plasma Pharmacokinetic Parameters of M3 7

Supplemental Table S4. CL-001 SAD (Part 1): Statistical Analysis of the Natural Log-Transformed Systemic Exposure of TBAJ-876 Comparing Single Doses of 100 mg TBAJ-876 Oral Suspension under Fed (Test) and Fasted Conditions (Reference) 9

Supplemental Table S5. CL-001 SAD (Part 1): Statistical Analysis of the Natural Log-Transformed Systemic Exposure of M3 Comparing Single Doses of 100 mg TBAJ-876 Oral Suspension under Fed (Test) and Fasted Conditions (Reference) 9

Supplemental Table S6. CL-001 rBA (Part 3): Statistical Analysis of the Natural Log-Transformed Systemic Exposure of TBAJ-876 Comparing Single Doses of 100 mg TBAJ-876 Tablets Administered under Fed (Test, Group 2) and Fasted Conditions (Reference, Group 1) 10

Supplemental Table S7. CL-001 rBA (Part 3): Statistical Analysis of the Natural Log-Transformed Systemic Exposure of M3 Comparing Single Doses of 100 mg TBAJ-876 Tablets Administered under Fed (Test, Group 2) and Fasted Conditions (Reference, Group 1) 10

Supplemental Table S8. CL-001 rBA (Part 3): Statistical Analysis of the Natural Log-Transformed Systemic Exposure of TBAJ-876 Comparing Single Doses of 100 mg TBAJ-876 Tablets Administered under Fasted Conditions as 4 x 25 mg Tablets (Test, Group 3) and as 1 x 100 mg Tablet (Reference, Group 1) 11

Supplemental Table S9. CL-001 rBA (Part 3): Statistical Analysis of the Natural Log-Transformed Systemic Exposure of M3 Comparing Single Doses of 100 mg TBAJ-876 Tablets Administered under Fasted Conditions as 4 x 25 mg Tablets (Test, Group 3) and as 1 x 100 mg Tablet (Reference, Group 1) 11

Supplemental Table S10. CL-001: Plasma Pharmacokinetic Parameters of Tablets (rBA, Part 3) versus Oral Suspension (SAD, Part 1) 12

Supplemental Table S11. CL-001 MAD (Part 2): Tukey's Multiple Comparison Test for Trough Concentrations of TBAJ-876 after Multiple, Once-Daily Doses of 25 mg (Cohort 1), 75 mg (Cohort 2), and 200 mg (Cohort 3) TBAJ-876 Oral Suspension 13

Supplemental Table S12. CL-001 MAD (Part 2): Plasma Pharmacokinetic Parameters of TBAJ-876 Day 14 14

Supplemental Table S13. CL-001 MAD (Part 2): Plasma Pharmacokinetic Parameters of M2 Day 14 15

Supplemental Table S14. CL-001 MAD (Part 2): Plasma Pharmacokinetic Parameters of M3 Day 14 16

Supplemental Table S15. CL-001 SAD (Part 1): Assessment of Dose-Proportionality of TBAJ-876 Following Single Dose Administrations of TBAJ-876 Oral Suspension 17
Supplemental Table S16. CL-001 MAD (Part 2): Assessment of Dose-Proportionality of TBAJ-876 Following the First Dose Administration of TBAJ-876 Oral Suspension on Day 1 17
Supplemental Table S17. CL-001 MAD (Part 2): Assessment of Dose-Proportionality of M3 Following the First Dose Administration of TBAJ-876 Oral Suspension on Day 1 17
Supplemental Table S18. CL-001 MAD (Part 2): Assessment of Dose-Proportionality of TBAJ-876 Following Multiple, Once- Daily Administration of TBAJ-876 Oral Suspension over 14 Days (Day 14) 18
Supplemental Table S19. CL-001 MAD (Part 2): Assessment of Dose-Proportionality of M2 Following Multiple, Once-Daily Administration of TBAJ-876 Oral Suspension over 14 Days (Day 14) 18
Supplemental Table S20. CL-001 MAD (Part 2): Assessment of Dose-Proportionality of M3 Following Multiple, Once-Daily Administration of TBAJ-876 Oral Suspension over 14 Days (Day 14) 18
Supplemental Table S21. CL-002 DDI: Summary of Pharmacokinetic Parameters after 12 Days of Dosing – PK Population 19
Supplemental Table S22. CL-001: Summary of Treatment Emergent Adverse Events, All Three Parts 20
Supplemental Table S23. CL-002: Summary of Treatment Emergent Adverse Events 25
Supplemental Table S24. CL-001 Drug Concentration Measurement Times 27
Supplemental Figures 29
Figure S1 CL-001 MAD (Part 2): Comparing TBAJ-876 Mean Concentration Profiles Day 1 Versus Day 14 29
Figure S2 CL-001 MAD (Part 2): Comparing M3 Mean Concentration Profiles Day 1 Versus Day 14 30
Figure S3 CL-001 MAD (Part 3): Comparing M2 Mean Concentration Profiles Day 1 Versus Day 14 31

Supplemental Tables

Supplemental Table S1. CL-001 SAD (Part 1): Plasma Pharmacokinetic Parameters of M2

Parameter	Cohort 4: TBAJ-876 (100 mg) Fasted				Cohort 5: TBAJ-876 (200 mg)			
	n	Mean	SD	CV%	n	Mean	SD	CV%
T_{max} [a] (h)	7	12.0 (6.12-32.0)			6	8.01 (7.99-28.0)		
C_{max} (ng/mL)	7	1.54	0.138	8.98	6	2.62	0.705	27.0
$AUC_{0\text{-}24h}$ (h*ng/mL)	6	25.1	2.56	10.2	6	40.8	12.0	29.4
$AUC_{0\text{-}72h}$ (h*ng/mL)	5	83.6	7.62	9.11	6	119	28.0	23.5
AUC_{last} (h*ng/mL)	7	65.1	39.2	60.2	6	224	166	74.0
λz (h^{-1})	1	0.00462	NC	NC	2	0.00730	0.00320	43.9
$t_{1/2}$ (h)	1	150	NC	NC	2	105	46.1	43.9
T_{last} (h)	7	56.9	29.3	51.5	6	155	116	75.0
C_{last} (ng/mL)	7	1.14	0.171	14.9	6	1.12	0.111	9.98
**Parameter**	**n**	**Cohort 6: TBAJ-876 (400 mg)**		**CV%**	**n**	**Cohort 7: TBAJ-876 (800 mg)**		**CV%**
		**Mean**	**SD**			**Mean**	**SD**	
T_{max} [a] (h)	6	12.0 (6.05-16.0)			6	12.0 (10.0-36.0)		
C_{max} (ng/mL)	6	5.03	1.44	28.6	6	8.60	5.22	60.7
$AUC_{0\text{-}24h}$ (h*ng/mL)	6	85.0	23.3	27.4	6	136	70.3	51.6
$AUC_{0\text{-}72h}$ (h*ng/mL)	6	251	62.7	25.0	6	406	201	49.5
AUC_{last} (h*ng/mL)	6	731	324	44.3	6	1040	581	55.7
λz (h^{-1})	5	0.00361	0.000915	25.4	5	0.00409	0.00149	36.4
$t_{1/2}$ (h)	5	203	52.9	26.1	5	187	63.0	33.6
T_{last} (h)	6	315	109	34.5	6	264	74.3	28.1
C_{last} (ng/mL)	6	1.31	0.241	18.5	6	2.08	0.748	36.0

[a] T_{max} presented as median (range)
NC = Not calculated
Note: Only 1 quantifiable M2 concentration was reported for Cohorts 1 through 3. This concentration was excluded from PK analysis. Limited quantifiable M2 concentration-time data were available for only 1 subject after administration of 100 mg oral suspension under fed conditions.M2 AUC_{inf} was not determined due to AUC_{Extrap} >20% for all evaluable subjects.

Supplemental Table S2. CL-001 SAD (Part 1): Plasma Pharmacokinetic Parameters of TBAJ-876

**Parameter**	**n**	**<u>Cohort 1</u>: TBAJ-876 (10 mg)**		**CV%**	**n**	**<u>Cohort 2</u>: TBAJ-876 (25 mg)**		**CV%**
		**Mean**	**SD**			**Mean**	**SD**	
T_{max}^{a}(h)	6	6.00 (4.00-8.00)			6	5.99 (2.99-8.00)		
C_{max} (ng/mL)	6	18.8	5.99	31.8	6	54.3	20.4	37.5
$AUC_{0\text{-}24h}$ (h*ng/mL)	6	214	58.6	27.4	6	588	213	36.3
$AUC_{0\text{-}72h}$ (h*ng/mL)	6	277	72.0	26.0	6	765	276	36.1
AUC_{last} (h*ng/mL)	6	259	73.2	28.3	6	831	334	40.1
AUC_{inf} (h*ng/mL)	5	300	68.0	22.6	6	896	360	40.2
AUC_{Extrap} (%)	5	9.28	3.70	39.8	6	7.25	1.33	18.4
λz (h^{-1})	5	0.0530	0.0204	38.4	6	0.0217	0.00981	45.2
$t_{1/2}$ (h)	5	14.9	5.92	39.9	6	38.9	19.2	49.3
T_{last} (h)	6	44.0	6.20	14.1	6	113	36.3	32.2
C_{last} (ng/mL)	6	1.28	0.188	14.7	6	1.16	0.111	9.55
CL/F (L/h)	5	34.6	7.25	21.0	6	33.9	19.0	56.2
Vz/F (L)	5	764	407	53.3	6	1590	406	25.6
**Parameter**	**n**	**<u>Cohort 3</u>: TBAJ-876 (50 mg)**		**CV%**	**n**	**<u>Cohort 4</u>: TBAJ-876 (100 mg) Fasted**		
		**Mean**	**SD**			**Mean**	**SD**	**CV%**
T_{max}^{a} (h)	6	5.99 (5.99-6.07)			9	6.00 (5.00-6.12)		
C_{max} (ng/mL)	6	103	46.2	44.9	9	215	56.5	26.3
$AUC_{0\text{-}24h}$ (h*ng/mL)	6	1180	530	45.0	9	2250	634	28.2
$AUC_{0\text{-}72h}$ (h*ng/mL)	6	1560	720	46.1	9	3000	863	28.8
AUC_{last} (h*ng/mL)	6	2440	1190	49.0	9	5480	2110	38.5
AUC_{inf} (h*ng/mL)	1	721	NC	NC	2	4040	1120	27.8
AUC_{Extrap} (%)	1	7.89	NC	NC	2	9.30	4.98	53.6
λz (h^{-1})	2	0.00920	0.0129	140	4	0.0107	0.0194	182
$t_{1/2}$ (h)	2	3340	4670	140	4	1190	1340	113
T_{last} (h)	6	560	216	38.5	9	1100	606	54.9
C_{last} (ng/mL)	6	1.48	0.578	39.1	9	2.40	1.99	83.1
CL/F (L/h)	1	69.4	NC	NC	2	25.8	7.16	27.8
Vz/F (L)	1	3790	NC	NC	2	5130	6170	120

Parameter	n	Cohort 5: TBAJ-876 (200 mg) Mean	SD	CV%	n	Cohort 6: TBAJ-876 (400 mg) Mean	SD	CV%
T_{max} [a](h)	6	6.00 (3.00-6.02)			6	6.00 (5.00-6.02)		
C_{max} (ng/mL)	6	506	92.7	18.3	6	780	176	22.6
AUC_{0-24h} (h*ng/mL)	6	4990	929	18.6	6	7190	1920	26.7
AUC_{0-72h} (h*ng/mL)	6	6490	1220	18.8	6	9780	2800	28.7
AUC_{last} (h*ng/mL)	6	13400	3910	29.2	6	22100	6970	31.6
AUC_{inf} (h*ng/mL)	2	14500	428	2.95	1	30500	NC	NC
AUC_{Extrap} (%)	2	11.0	2.29	20.8	1	18.8	NC	NC
λz (h^{-1})	3	0.00104	0.000392	37.8	5	0.000533	0.000229	42.8
$t_{1/2}$ (h)	3	739	283	38.3	5	1880	1730	92.0
T_{last} (h)	6	1500	415	27.8	6	1490	412	27.6
C_{last} (ng/mL)	6	3.02	1.04	34.5	6	5.25	0.958	18.2
CL/F (L/h)	2	13.8	0.407	2.95	1	13.1	NC	NC
Vz/F (L)	2	11700	2720	23.3	1	18400	NC	NC

Parameter	Cohort 7: TBAJ-876 (800 mg)			
	n	Mean	SD	CV%
T_{max} [a](h)	6		5.99 (4.01-6.00)	
C_{max} (ng/mL)	6	1320	393	29.8
AUC_{0-24h} (h*ng/mL)	6	12400	3010	24.2
AUC_{0-72h} (h*ng/mL)	6	16400	3820	23.3
AUC_{last} (h*ng/mL)	6	22400	5190	23.2
λz (h^{-1})	4	0.00123	0.000595	48.5
$t_{1/2}$ (h)	4	672	303	45.1
T_{last} (h)	6	316	10.2	3.22
C_{last} (ng/mL)	6	19.5	7.53	38.7

[a]T_{max} presented as median (range)

Note: AUC_{inf} was not reported for Cohort 7 due to AUC_{Extrap} >20% for all evaluable subjects.

Supplemental Table S3. CL-001 SAD (Part 1): Plasma Pharmacokinetic Parameters of M3

Parameter	n	Cohort 2: TBAJ-876 (25 mg)		CV%	n	Cohort 3: TBAJ-876 (50 mg)		CV%
		Mean	SD			Mean	SD	
T_{max} [a] (h)	5	12.0 (7.99-12.1)			6	12.0 (8.10-12.0)		
C_{max} (ng/mL)	5	2.85	0.806	28.3	6	4.08	1.20	29.5
AUC_{0-24h} (h*ng/mL)	5	42.1	14.6	34.7	6	66.6	18.3	27.4
AUC_{0-72h} (h*ng/mL)	5	115	35.6	31.0	6	188	32.3	17.2
AUC_{last} (h*ng/mL)	5	129	67.2	52.1	6	434	152	34.9
λz (h^{-1})	3	0.00984	0.00376	38.3	5	0.00409	0.00286	70.0
$t_{1/2}$ (h)	3	78.5	32.4	41.3	5	403	531	132
T_{last} (h)	5	81.6	25.6	31.3	6	260	113	43.5
C_{last} (ng/mL)	5	1.11	0.0677	6.08	6	1.10	0.134	12.2
**Parameter**	**n**	**Cohort 4: TBAJ-876 (100 mg) Fasted**			**n**	**Cohort 5: TBAJ-876 (200 mg)**		**CV%**
		**Mean**	**SD**	**CV%**		**Mean**	**SD**	
T_{max} [a] (h)	9	12.0 (6.12-16.0)			6	10.0 (7.99-16.0)		
C_{max} (ng/mL)	9	10.5	2.02	19.2	6	19.1	4.80	25.1
AUC_{0-24h} (h*ng/mL)	9	162	29.1	18.0	6	306	81.9	26.8
AUC_{0-72h} (h*ng/mL)	9	429	83.1	19.4	6	787	153	19.5
AUC_{last} (h*ng/mL)	9	1200	468	38.8	6	2940	656	22.3
AUC_{inf} (h*ng/mL)	1	1400	NC	NC	3	3610	940	26.0
AUC_{Extrap} (%)	1	19.6	NC	NC	3	14.3	1.26	8.84
λz (h^{-1})	9	0.00544	0.00942	173	6	0.00201	0.000706	35.1
$t_{1/2}$ (h)	9	357	334	93.5	6	383	135	35.3
T_{last} (h)	9	442	173	39.1	6	764	180	23.5
C_{last} (ng/mL)	9	1.40	0.756	54.1	6	1.55	0.406	26.2

Parameter	n	Cohort 6: TBAJ-876 (400 mg) Mean	SD	CV%	n	Cohort 7: TBAJ-876 (800 mg) Mean	SD	CV%
T_{max} [a] (h)	6	12.0 (6.05-12.0)			6	11.0 (8.01-12.0)		
C_{max} (ng/mL)	6	31.0	8.04	25.9	6	49.1	21.2	43.2
AUC_{0-24h} (h*ng/mL)	6	511	111	21.6	6	821	368	44.8
AUC_{0-72h} (h*ng/mL)	6	1330	260	19.6	6	2110	875	41.5
AUC_{last} (h*ng/mL)	6	6410	1500	23.5	6	5370	2190	40.8
AUC_{inf} (h*ng/mL)	6	7250	1390	19.1	0	NC	NC	NC
AUC_{Extrap} (%)	6	12.2	4.12	33.7	0	NC	NC	NC
λz (h^{-1})	6	0.00166	0.000531	32.1	4	0.00282	0.000641	22.7
$t_{1/2}$ (h)	6	454	136	30.0	4	256	62.9	24.5
T_{last} (h)	6	1380	393	28.6	6	316	10.2	3.22
C_{last} (ng/mL)	6	1.37	0.464	34.0	6	9.54	4.34	45.4

[a] T_{max} presented as median (range)

NC = Not calculated

Note: Only 2 quantifiable M3 concentrations was reported for Cohort 1. These limited concentration-time data were excluded from PK analysis. M3 AUC_{inf} was not determined for Cohorts 2, 3, and 7 due to AUC_{Extrap} >20% for all evaluable subjects.

Supplemental Table S4. CL-001 SAD (Part 1): Statistical Analysis of the Natural Log-Transformed Systemic Exposure of TBAJ-876 Comparing Single Doses of 100 mg TBAJ-876 Oral Suspension under Fed (Test) and Fasted Conditions (Reference)

Dependent Variable	Geometric Mean[a] Test (Fed)	Ref (Fasted)	Ratio (%)[b] (Test/Ref)	90% CI[c] Lower	Upper	Power[d]	ANOVA CV%[e]
$\ln(C_{max})$	408	208	195.83	153.76	249.40	0.4493	30.96
$\ln(AUC_{0\text{-}24h})$	3480	2180	159.97	126.79	201.84	0.4742	29.71
$\ln(AUC_{0\text{-}72h})$	4610	2890	159.15	125.84	201.27	0.4677	30.03
$\ln(AUC_{last})$	6960	5170	134.66	103.91	174.51	0.4082	33.30
$\ln(AUC_{inf})$	6710	3960	169.48	73.64	390.05	0.1159	29.14

[a] Geometric Mean for 100 mg TBAJ-876 oral suspension under Fed (Test) and Fasted (Ref) conditions based on Least Squares Mean of log-transformed parameter values

[b] Ratio(%) = 100 x Geometric Mean (Test)/Geometric Mean (Ref)

[c] 90% Confidence Interval

[d.] 1 – β error, based on 0.05 α error rate

[e] $100 \times (\exp(\text{MSResidual})-1)^{0.5}$

Note: AUC_{inf} data were not reported for most subjects due to AUC_{Extrap} >20%.

Supplemental Table S5. CL-001 SAD (Part 1): Statistical Analysis of the Natural Log-Transformed Systemic Exposure of M3 Comparing Single Doses of 100 mg TBAJ-876 Oral Suspension under Fed (Test) and Fasted Conditions (Reference)

Dependent Variable	Geometric Mean[a] Test (Fed)	Ref (Fasted)	Ratio (%)[b] (Test/Ref)	90% CI[c] Lower	Upper	Power[d]	ANOVA CV%[e]
$\ln(C_{max})$	4.74	10.4	45.76	35.81	58.47	0.4410	31.40
$\ln(AUC_{0\text{-}24h})$	79.9	160	50.01	38.83	64.41	0.4220	32.47
$\ln(AUC_{0\text{-}72h})$	235	423	55.56	43.46	71.03	0.4396	31.47
$\ln(AUC_{last})$	658	1100	60.06	38.14	94.59	0.2037	61.74

[a] Geometric Mean for 100 mg TBAJ-876 oral suspension under Fed (Test) and Fasted (Ref) conditions based on Least Squares Mean of log-transformed parameter values

[b] Ratio (%) = 100 x Geometric Mean (Test)/Geometric Mean (Ref)

[c] 90% Confidence Interval

[d] 1 – β error, based on 0.05 α error rate

[e] $100 \times (\exp(\text{MSResidual})-1)^{0.5}$

Note: AUC_{inf} values were not reported for most subjects due to AUC_{Extrap} >20%.

Supplemental Table S6. CL-001 rBA (Part 3): Statistical Analysis of the Natural Log-Transformed Systemic Exposure of TBAJ-876 Comparing Single Doses of 100 mg TBAJ-876 Tablets Administered under Fed (Test, Group 2) and Fasted Conditions (Reference, Group 1)

**Dependent Variable**	**GeoMean [a] Fed Test**	**GeoMean [a] Fasted Ref**	**Ratio (%)[b] (Fed/Fasted)**	**90% CI [c] Lower**	**90% CI [c] Upper**	**Power[d]**	**ANOVA[e] CV%**
$\ln(C_{max})$	422	182	231.53	174.55	307.11	0.3630	37.67
$\ln(AUC_{0\text{-}24h})$	3940	2130	184.56	142.08	239.74	0.4033	34.72
$\ln(AUC_{last})$	7270	3760	193.61	148.16	252.99	0.3910	35.55
$\ln(AUC_{inf})$	8570	3520	243.39	164.74	359.60	0.2354	36.28

[a] Geometric Mean based on Least Squares Mean

[b] Ratio (%) = 100 x Geometric Mean (Test)/Geometric Mean (Ref)

[c] 90% Confidence Interval

[d] 1 – β error, based on 0.05 α error rate

[e] 100 x $(\exp(\text{MSResidual})-1)^{0.5}$

Supplemental Table S7. CL-001 rBA (Part 3): Statistical Analysis of the Natural Log-Transformed Systemic Exposure of M3 Comparing Single Doses of 100 mg TBAJ-876 Tablets Administered under Fed (Test, Group 2) and Fasted Conditions (Reference, Group 1)

**Dependent Variable**	**GeoMean [b] Fed Test**	**GeoMean [b] Fasted Ref**	**Ratio (%)[e] (Fed/Fasted)**	**90% CI [f] Lower**	**90% CI [f] Upper**	**Power[h]**	**ANOVA[e] CV%**
$\ln(C_{max})$	6.34	7.87	80.57	55.87	116.18	0.2595	49.96
$\ln(AUC_{0\text{-}24h})$	96.2	122	79.10	53.50	116.94	0.2401	53.80
$\ln(AUC_{last})$	920	723	127.30	82.15	197.25	0.2118	61.29

[a] Geometric Mean based on Least Squares Mean

[b] Ratio (%) = 100 x Geometric Mean (Test)/Geometric Mean (Ref)

[c] 90% Confidence Interval

[d] 1 – β error, based on 0.05 α error rate

[e] 100 x $(\exp(\text{MSResidual})-1)^{0.5}$

Note: AUC_{inf} was not reported due to AUC_{Extrap} >20% for most subjects

Supplemental Table S8. CL-001 rBA (Part 3): Statistical Analysis of the Natural Log-Transformed Systemic Exposure of TBAJ-876 Comparing Single Doses of 100 mg TBAJ-876 Tablets Administered under Fasted Conditions as 4 x 25 mg Tablets (Test, Group 3) and as 1 x 100 mg Tablet (Reference, Group 1)

Dependent Variable	GeoMean [b] Test	GeoMean [b] Ref	Ratio (%)[c] (Test/Ref)	90% CI [f] Lower	90% CI [f] Upper	Power[h]	ANOVA[e] CV%
$\ln(C_{max})$	182	182	99.75	72.56	137.13	0.3096	42.83
$\ln(AUC_{0\text{-}24h})$	2340	2130	109.43	81.30	147.30	0.3391	39.77
$\ln(AUC_{last})$	4100	3760	109.26	80.48	148.32	0.3265	41.00
$\ln(AUC_{inf})$	4120	3520	117.07	73.27	187.06	0.1931	44.17

[a] Geometric Mean based on Least Squares Mean

[b] Ratio (%) = 100 x Geometric Mean (Test)/Geometric Mean (Ref)

[c] 90% Confidence Interval

[d] 1 – β error, based on 0.05 α error rate

[e] 100 x $(\exp(\text{MSResidual})-1)^{0.5}$

Supplemental Table S9. CL-001 rBA (Part 3): Statistical Analysis of the Natural Log-Transformed Systemic Exposure of M3 Comparing Single Doses of 100 mg TBAJ-876 Tablets Administered under Fasted Conditions as 4 x 25 mg Tablets (Test, Group 3) and as 1 x 100 mg Tablet (Reference, Group 1)

Dependent Variable	GeoMean [a] Test	GeoMean [a] Ref	Ratio (%)[b] (Test/Ref)	90% CI [c] Lower	90% CI [c] Upper	Power[d]	ANOVA[e] CV%
$\ln(C_{max})$	6.81	7.87	86.44	55.35	135.00	0.2079	62.57
$\ln(AUC_{0\text{-}24h})$	106	122	87.33	54.39	140.23	0.1957	67.23
$\ln(AUC_{last})$	677	723	93.64	49.94	175.60	0.1542	96.41

[a] Geometric Mean based on Least Squares Mean

[b] Ratio (%) = 100 x Geometric Mean (Test)/Geometric Mean (Ref)

[c] 90% Confidence Interval

[d] 1 – β error, based on 0.05 α error rate

[e] 100 x $(\exp(\text{MSResidual})-1)^{0.5}$

Note: AUCinf was not reported due to AUCExtrap >20% for most subjects

Supplemental Table S10. CL-001: Plasma Pharmacokinetic Parameters of Tablets (rBA, Part 3) versus Oral Suspension (SAD, Part 1)

Analyte	Formulation	Fasted			Fed		
		T_{max} (h)	C_{max} (ng/mL)	$AUC_{0\text{-}24h}$ (h*ng/mL)	T_{max} (h)	C_{max} (ng/mL)	$AUC_{0\text{-}24h}$ (h*ng/mL)
TBAJ-876	Suspension	6.00	215	2250	4.00	429	3640
TBAJ-876	100 mg Tablet	6.00	197	2310	5.00	441	4040
TBAJ-876	4x25 mg Tablets	6.00	194	2460	-	-	-
M2	Suspension	12.0	1.54	25.1	-	-	-
M2	100 mg Tablet	12.0	1.78	28.7	-	-	-
M2	4x25 mg Tablets	12.0	1.40	19.7	-	-	-
M3	Suspension	12.0	10.5	162	12.0	5.04	85.5
M3	100 mg Tablet	12.0	9.37	147	16.0	6.49	98.3
M3	4x25 mg Tablets	12.0	7.47	117	-	-	-

Values shown are medians for T_{max}, arithmetic means for C_{max} and $AUC_{0\text{-}24h}$.
Results for M2 under fed conditions not shown because n = 1.

Supplemental Table S11. CL-001 MAD (Part 2): Tukey's Multiple Comparison Test for Trough Concentrations of TBAJ-876 after Multiple, Once-Daily Doses of 25 mg (Cohort 1), 75 mg (Cohort 2), and 200 mg (Cohort 3) TBAJ-876 Oral Suspension

Cohort	Concentration (ng/mL)	Study Day	2	3	4	5	6	7	8	9	10	11	12	13	14
Cohort 1	15.484	2	.	.	.	.	.	.	.	.	.	.	.	.	.
	20.411	3	0.0553	.	.	.	.	.	.	.	.	.	.	.	.
	22.389	4	0.0006	0.9789	.	.	.	.	.	.	.	.	.	.	.
	25.811	5	<.0001	0.0213	0.4980	.	.	.	.	.	.	.	.	.	.
	26.367	6	<.0001	0.0062	0.2616	1.0000	.	.	.	.	.	.	.	.	.
	25.122	7	<.0001	0.0823	0.8109	1.0000	0.9997	.	.	.	.	.	.	.	.
	28.744	8	<.0001	<.0001	0.0024	0.7292	0.9184	0.4050	.	.	.	.	.	.	.
	31.522	9	<.0001	<.0001	<.0001	0.0108	0.0354	0.0021	0.7939	.	.	.	.	.	.
	32.100	10	<.0001	<.0001	<.0001	0.0028	0.0103	0.0005	0.5301	1.0000	.	.	.	.	.
	30.678	11	<.0001	<.0001	<.0001	0.0619	0.1610	0.0153	0.9825	1.0000	0.9989	.	.	.	.
	33.367	12	<.0001	<.0001	<.0001	<.0001	0.0004	<.0001	0.0963	0.9881	0.9997	0.8272	.	.	.
	35.422	13	<.0001	<.0001	<.0001	<.0001	<.0001	<.0001	0.0010	0.2900	0.5462	0.0775	0.9714	.	.
	36.100	14	<.0001	<.0001	<.0001	<.0001	<.0001	<.0001	0.0002	0.1040	0.2539	0.0204	0.8109	1.0000	.

Supplemental Table S12. CL-001 MAD (Part 2): Plasma Pharmacokinetic Parameters of TBAJ-876 Day 14

Parameter	Cohort 1: TBAJ-876 (25 mg)				Cohort 2: TBAJ-876 (75 mg)				Cohort 3 TBAJ-876 (200 mg)			
	n	Mean	SD	CV%	n	Mean	SD	CV%	n	Mean	SD	CV%
T_{max} [a] (h)	9	5.00 (2.00-5.01)			9	5.00 (2.07-5.01)			9	5.00 (3.01-5.02)		
C_{max} (ng/mL)	9	148	47.0	31.8	9	401	122	30.3	9	1160	502	43.4
AUC_{0-24h} (h*ng/mL)	9	1670	480	28.7	9	4240	1110	26.1	9	12900	5780	44.7
C_{min} (ng/mL)	9	33.7	10.3	30.6	9	77.9	16.8	21.6	9	243	84.5	34.8
C_{24} (ng/mL)	9	34.1	8.27	24.3	9	81.9	25.8	31.4	9	266	93.6	35.1
C_{avg} (ng/mL)	9	69.7	20.0	28.7	9	177	46.1	26.1	9	539	241	44.7
RC_{max}	9	1.60	0.387	24.3	9	1.27	0.206	16.2	9	1.38	0.463	33.5
R_{C24}	9	2.85	0.503	17.7	9	2.28	0.619	27.2	9	3.61	0.892	24.7
R_{AUC}	9	1.78	0.206	11.6	9	1.41	0.183	13.0	9	1.75	0.452	25.8

[a] T_{max} presented as median (range)

Supplemental Table S13. CL-001 MAD (Part 2): Plasma Pharmacokinetic Parameters of M2 Day 14

Parameter	Cohort 1: TBAJ-876 (25 mg)				Cohort 2: TBAJ-876 (75 mg)				Cohort 3 TBAJ-876 (200 mg)			
	n	Mean	SD	CV%	n	Mean	SD	CV%	n	Mean	SD	CV%
T_{max} [a] (h)	9	4.04 (0.987-16.0)			9	4.01 (0.499-5.00)			9	5.00 (3.01-8.00)		
C_{max} (ng/mL)	9	2.01	0.681	33.8	9	6.49	1.65	25.4	9	16.0	5.30	33.2
AUC_{0-24h} (h*ng/mL)	9	39.9	12.1	30.3	9	124	32.9	26.5	9	309	97.9	31.7
C_{min} (ng/mL)	9	1.28	0.607	47.6	9	4.16	1.17	28.2	9	10.6	3.23	30.5
C_{24} (ng/mL)	9	1.63	0.467	28.6	9	4.50	1.48	33.0	9	12.5	3.99	31.9
C_{avg} (ng/mL)	9	1.66	0.504	30.3	9	5.17	1.37	26.5	9	12.9	4.08	31.7
RC_{max}	0	NC	NC	NC	0	NC	NC	NC	5	9.59	3.22	33.6
R_{C24}	0	NC	NC	NC	0	NC	NC	NC	5	11.7	1.62	13.8
R_{AUC}	0	NC	NC	NC	0	NC	NC	NC	5	12.5	2.98	23.9

[a] T_{max} presented as median (range)

NC = Not Calculated

Supplemental Table S14. CL-001 MAD (Part 2): Plasma Pharmacokinetic Parameters of M3 Day 14

Parameter	Cohort 1: TBAJ-876 (25 mg)				Cohort 2: TBAJ-876 (75 mg)				Cohort 3 TBAJ-876 (200 mg)			
	n	Mean	SD	CV%	n	Mean	SD	CV%	n	Mean	SD	CV%
T_{max} [a] (h)	9	5.00 (2.00-16.0)			9	5.00 (0.499-12.0)			9	5.00 (3.01-8.00)		
C_{max} (ng/mL)	9	10.6	3.96	37.5	9	35.2	7.70	21.9	9	85.8	27.2	31.7
AUC_{0-24h} (h*ng/mL)	9	209	77.2	36.9	9	649	133	20.5	9	1620	459	28.3
C_{min} (ng/mL)	9	7.22	2.80	38.9	9	21.5	5.27	24.5	9	55.7	15.2	27.3
C_{24} (ng/mL)	9	8.25	3.04	36.9	9	23.3	5.35	22.9	9	64.4	19.1	29.7
C_{avg} (ng/mL)	9	8.71	3.22	36.9	9	27.0	5.53	20.5	9	67.6	19.1	28.3
RC_{max}	6	9.63	0.614	6.37	9	9.65	3.03	31.4	9	8.69	2.72	31.3
R_{C24}	1	7.87	ND	ND	3	7.54	0.544	7.22	7	8.41	1.64	19.5
R_{AUC}	1	11.0	ND	ND	3	9.91	2.47	24.9	7	9.16	2.09	22.8

[a]T_{max} presented as median (range)

ND = Not determinable

Supplemental Table S15. CL-001 SAD (Part 1): Assessment of Dose-Proportionality of TBAJ-876 Following Single Dose Administrations of TBAJ-876 Oral Suspension

Dependent Variable	Model Variable	Estimate (β1)	Lower CI[a]	Upper CI[a]
ln(C_{max})	ln(Dose)	0.9921	0.9294	1.0548
ln($AUC_{0\text{-}24h}$)	ln(Dose)	0.9380	0.8779	0.9982
ln($AUC_{0\text{-}72h}$)	ln(Dose)	0.9446	0.8846	1.0047
ln(AUC_{last})	ln(Dose)	1.0919	1.0029	1.1809
ln(AUC_{inf})	ln(Dose)	1.2443	1.0876	1.4010

Cohort 1: TBAJ-876 10 mg; Cohort 2: TBAJ-876 25 mg; Cohort 3: TBAJ-876 50 mg; Cohort 4: TBAJ-876 100 mg (fasted); Cohort 5: TBAJ-876 200 mg; Cohort 6: TBAJ-876 400 mg; Cohort 7: 800 mg

Power Model: ln(PK)=ln(β0)+β1*ln(Dose)+e, where PK is the pharmacokinetic parameter tested, ln(β_0) is the y- intercept, β_1 is the slope, and e is an error term

[a] 90% confidence intervals (Lower and Upper)

Supplemental Table S16. CL-001 MAD (Part 2): Assessment of Dose-Proportionality of TBAJ-876 Following the First Dose Administration of TBAJ-876 Oral Suspension on Day 1

Dependent Variable	Model Variable	Estimate (β1)	Lower CI[a]	Upper CI[a]
ln(C_{max})	ln(Dose)	1.0528	0.9172	1.1885
ln($AUC_{0\text{-}24h}$)	ln(Dose)	0.9822	0.8795	1.0848

Cohort 1: TBAJ-876 25 mg; Cohort 2: TBAJ-876 75 mg; Cohort 3: TBAJ-876 200 mg

Power Model: ln(PK)=ln(β_0)+β1*ln(Dose)+e, where PK is the pharmacokinetic parameter tested, ln(β_0) is the y-intercept, β1 is the slope, and e is an error term

[a] 90% confidence intervals (Lower and Upper)

Supplemental Table S17. CL-001 MAD (Part 2): Assessment of Dose-Proportionality of M3 Following the First Dose Administration of TBAJ-876 Oral Suspension on Day 1

Dependent Variable	Model Variable	Estimate (β1)	Lower CI[a]	Upper CI[a]
ln(C_{max})	ln(Dose)	0.9778	0.8131	1.1425
ln($AUC_{0\text{-}24h}$)	ln(Dose)	0.9919	0.6433	1.3405

Cohort 1: TBAJ-876 25 mg; Cohort 2: TBAJ-876 75 mg; Cohort 3: TBAJ-876 200 mg

Power Model: ln(PK)=ln(β_0)+β1*ln(Dose)+e, where PK is the pharmacokinetic parameter tested, ln(β0) is the y-intercept, β1 is the slope, and e is an error term

[a] 90% confidence intervals (Lower and Upper)

Supplemental Table S18. CL-001 MAD (Part 2): Assessment of Dose-Proportionality of TBAJ-876 Following Multiple, Once- Daily Administration of TBAJ-876 Oral Suspension over 14 Days (Day 14)

Dependent Variable	Model Variable	Estimate (β1)	Lower CI[a]	Upper CI[a]
$\ln(C_{max})$	ln(Dose)	0.9735	0.8430	1.1039
$\ln(AUC_{0\text{-}24h})$*	ln(Dose)	0.9639	0.8379	1.0900

Cohort 1: TBAJ-876 25 mg; Cohort 2: TBAJ-876 75 mg; Cohort 3: TBAJ-876 200 mg

Power Model: $\ln(PK)=\ln(\beta_0)+\beta1*\ln(Dose)+e$, where PK is the pharmacokinetic parameter tested, $\ln(\beta_0)$ is the y-intercept, β1 is the slope, and e is an error term

[a] 90% confidence intervals (Lower and Upper)

*$AUC_{0\text{-}24h}$ = AUC_{tau} where tau = 24 hours

Supplemental Table S19. CL-001 MAD (Part 2): Assessment of Dose-Proportionality of M2 Following Multiple, Once-Daily Administration of TBAJ-876 Oral Suspension over 14 Days (Day 14)

Dependent Variable	Model Variable	Estimate (β1)	Lower CI[a]	Upper CI[a]
$\ln(C_{max})$	ln(Dose)	1.0043	0.8870	1.1217
$\ln(AUC_{0\text{-}24h})$*	ln(Dose)	0.9864	0.8785	1.0942

Cohort 1: TBAJ-876 25 mg; Cohort 2: TBAJ-876 75 mg; Cohort 3: TBAJ-876 200 mg

Power Model: $\ln(PK)=\ln(\beta_0)+\beta1*\ln(Dose)+e$, where PK is the pharmacokinetic parameter tested, $\ln(\beta_0)$ is the y-intercept, β1 is the slope, and e is an error term

[a] 90% confidence intervals (Lower and Upper)

*$AUC_{0\text{-}24h}$ = AUC_{tau} where tau = 24 hours

Supplemental Table S20. CL-001 MAD (Part 2): Assessment of Dose-Proportionality of M3 Following Multiple, Once-Daily Administration of TBAJ-876 Oral Suspension over 14 Days (Day 14)

Dependent Variable	Model Variable	Estimate (β1)	Lower CI[a]	Upper CI[a]
$\ln(C_{max})$	ln(Dose)	1.0199	0.8956	1.1443
$\ln(AUC_{0\text{-}24h})$*	ln(Dose)	0.9992	0.8856	1.1128

Cohort 1: TBAJ-876 25 mg; Cohort 2: TBAJ-876 75 mg; Cohort 3: TBAJ-876 200 mg

Power Model: $\ln(PK)=\ln(\beta_0)+\beta1*\ln(Dose)+e$, where PK is the pharmacokinetic parameter tested, $\ln(\beta_0)$ is the y- intercept, β1 is the slope, and e is an error term

[a] 90% confidence intervals (Lower and Upper)

*$AUC_{0\text{-}24h}$ = AUC_{tau} where tau = 24 hours

Supplemental Table S21. CL-002 DDI: Summary of Pharmacokinetic Parameters after 12 Days of Dosing – PK Population

Parameter	Statistics	TBAJ-876	M2	M3	Sum of TBAJ-876 and M3
AUC0-24 (ng.hr/mL)	n	26	26	26	26
	Mean (SD)	14620 (5531)	372 (110)	1971 (543)	16591 (5644)
	Median	13847	354.7	1905	15679
	Geometric mean	13899	356	1900	15889
	CV (%)	31.6	31.3	28.3	29.4
	Minimum, maximum	7180, 37499	184, 592	1039, 3121	8570, 39426
Cmax (ng/mL)	n	26	26	26	26
	Mean (SD)	1224 (513)	19 (6)	101 (26)	1317 (521)
	Median	1105	18.1	101.1	1191
	Geometric mean	1151	18	98	1246
	CV (%)	34.3	32.7	28.0	32.8
	Minimum, maximum	696, 3230	10, 31	50, 154	767, 3339
Tmax (hr)	n	26	26	26	26
	Median	5.0	6.0	6.0	5.0
	Minimum, maximum	3, 7	3, 16	3, 16	3, 7
Ctrough (ng/mL)	n	26	26	26	26
	Mean (SD)	293 (110)	13 (4)	69 (24)	361 (119)
	Median	272.5	12.6	65.0	346.9
	Geometric mean	276	12	65	344
	CV (%)	35.2	36.3	34.7	32.4
	Minimum, maximum	131, 693	6, 24	37, 118	174, 749

SD=Standard deviation, CV=Coefficient of variation (calculated as 100*SQRT(EXP(VAR) – 1) with VAR= the variance of the log-transformed values).

Supplemental Table S22. CL-001: Summary of Treatment Emergent Adverse Events, All Three Parts

Parameter	Statistic	No. of events/subjects on TBAJ-876 (n=112)	No. of events/subjects on placebo (n=25)	No. of events/subjects total (with placebo) (n=137)
All TEAEs	Number of Events	79	45	124
	Number of Subjects, n (%)	42 (38%)	16 (64%)	58 (42%)
Treatment-Related TEAEs	Number of Events	32	19	51
	Number of Subjects, n (%)	20 (18%)	10 (40%)	30 (22%)
Severe TEAEs	Number of Events	1	1	2
	Number of Subjects, n (%)	1 (0.89%)	1 (4%)	2 (1.5%)
Serious TEAEs	Number of Events	0	0	0
	Number of Subjects, n (%)	0 (0.0%)	0 (0.0%)	0 (0.0%)
TEAEs Leading to Study Discontinuation	Number of Events	0	0	0
	Number of Subjects, n (%)	0 (0.0%)	0 (0.0%)	0 (0.0%)
TEAEs Leading to Withdrawal of Study Drug	Number of Events	0	1	1
	Number of Subjects, n (%)	0 (0.0%)	1 (4.0%)	1 (0.73%)
TEAEs Leading to Death	Number of Events	0	0	0
	Number of Subjects, n (%)	0 (0.0%)	0 (0.0%)	0 (0.0%)
Cardiac disorders	n (%) of subjects	4 (3.6%)	2 (8.0%)	6 (4.4%)
Postural orthostatic tachycardia syndrome		2	1	3
		1	1	2
Tachycardia		1	0	1

Ventricular Tachycardia				
Gastrointestinal disorders	n (%) of subjects	14 (12.5%)	6 (24%)	20 (15%)
Diarrhea		2	0	2
Constipation		2	3	5
Nausea		6	1	7
Vomiting		2	1	3
Abdominal Pain		3	1	4
Gastroesophageal Reflux Disease		2	0	2
Abnormal faeces		1	0	1
Dry mouth		1	0	1
Gastrointestinal sounds abnormal		1	0	1
General disorders and administration site conditions	n (%) of subjects	4 (3.6%)	1 (4%)	5(3.6%)
Pyrexia		0	1	1
Fatigue		2	0	2
Vessel puncture site haemorrhage		1	0	1
Feeling hot		0	1	1
Non-cardiac chest pain		1	0	1
Infections and infestations	n (%) of subjects	0 (0.0%)	1 (4%)	1 (0.73%)
Covid-19		0	1	1
Investigations	n (%) of subjects	5 (4.4%)	2 (8%)	7 (5.1%)
Alanine aminotransferase increased		2	0	2
Lipase increased		2	2	4
Amylase increased		0	1	1
Blood creatine phosphokinase MB increased		1	0	1

Musculoskeletal and connective tissue disorders	n (%) of subjects	8 (7.1%)	1 (4%)	9 (6.5%)
Rhabdomyolysis		2	0	2
Arthralgia		1	1	2
Pain in extremity		2	1	3
Muscle spasms		2	0	2
Myalgia		2	0	2
Neck pain		1	0	1
Back pain		1	0	1
Nervous system disorders	n (%) of subjects	11 (9.8%)	8 (32%)	19 (14%)
Headache		7	6	13
Dizziness		3	2	5
Presyncope		1	0	1
Dysgeusia		1	0	1
Paraesthesia		0	1	1
Respiratory, thoracic, and mediastinal disorders	n (%) of subjects	2 (1.8%)	2 (8%)	4 (2.9%)
Cough		1	0	1
Dry throat		0	1	1
Hiccups		1	0	1
Nasal congestion		0	1	1
Throat tightness		1	0	1
Vascular disorders	n (%) of subjects	2 (1.8%)	0 (0.0%)	2 (1.5%)
Orthostatic hypotension		1	0	1
Flushing		1	0	1
Renal and urinary disorders	n (%) of subjects	1 (0.89%)	1 (4.0%)	2 (1.5%)
Proteinuria		1	0	1

Urinary hesitation		0	1	1
Immune system disorders	n (%) of subjects	0 (0.0%)	1 (4.0%)	1 (0.73%)
Allergy to arthropod bite		0	1	1
Psychiatric disorders	n (%) of subjects	1 (0.89%)	1 (4.0%)	2 (1.5%)
Stress		0	1	1
Insomnia		1	0	1
Reproductive system and breast disorders	n (%) of subjects	1 (0.89%)	3 (12%)	4 (2.9%)
Dysmenorrhoea		0	1	1
Menometrorrhagia		1	0	1
Menopausal symptoms		0	1	1
Vulvovaginal pruritus		0	1	1
Skin and subcutaneous tissue disorders	n (%) of subjects	6 (5.4%)	5 (20%)	11 (8.0%)
Dermatitis contact		4	4	8
Hand dermatitis		1	1	2
Hyperhidrosis		1	0	1
Pruritus		0	1	1
Rash pruritic		0	1	1
Eye disorders	n (%) of subjects	1 (0.89%)	1 (4.0%)	2 (1.5%)
Dry eye		1	0	1
Eye pain		0	1	1
Photophobia		0	1	1
Blood and lymphatic system disorders	n (%) of subjects	1 (0.89%)	0 (0.0%)	1 (0.73%)
Neutropenia		1	0	1
Ear and labyrinth disorders	n (%) of subjects	1 (0.89%)	0 (0.0%)	1 (0.73%)
Vertigo		1	0	1

TEAE=Treatment-emergent adverse event (with onset on or after Day 1). Adverse events coded using MedDRA version 23.0.
A treatment-related AE is any AE classified as possibly, probably, or certainly related to study drug.
Percentages are based on the number of subjects in the Safety Analysis Set by treatment group and overall.

Supplemental Table S23. CL-002: Summary of Treatment Emergent Adverse Events

Parameter	Statistic	No. Of Events/Subjects on TBAJ-876 (n=28)
All TEAEs	Number of Events	19
	Number of Subjects, n (%)	9 (32.1)
Treatment-Related TEAEs	Number of Events	6
	Number of Subjects, n (%)	5 (17.9)
Severe TEAEs	Number of Events	0
	Number of Subjects, n (%)	0
TEAEs Leading to Study Discontinuation	Number of Events	1
	Number of Subjects, n (%)	1 (3.6)
Cardiac disorders	n (%) of subjects	1 (3.6)
Atrioventricular block first degree		1 (3.6)
Gastrointestinal disorders	n (%) of subjects	1 (3.6)
Diarrhea		1 (3.6)
Oral discomfort		1 (3.6)
General disorders and administration site conditions	n (%) of subjects	2 (7.1)
Chest discomfort		1 (3.6)
Pyrexia		1 (3.6)
Infections and infestations	n (%) of subjects	1 (3.6)
Pneumonia		1 (3.6)
Investigations	n (%) of subjects	3 (10.7)
Alanine aminotransferase increased		1 (3.6)
Electrocardiogram PR interval elongation		1 (3.6)
Electrocardiogram ST segment elevation		1 (3.6)
Musculoskeletal and connective tissue disorders	n (%) of subjects	1 (3.6)
Flank pain		1 (3.6)
Nervous system disorders	n (%) of subjects	3 (10.7)
Headache		2 (7.1)
Lethargy		1 (3.6)

Respiratory, thoracic, and mediastinal disorders	n (%) of subjects	1 (3.6)
Cough		1 (3.6)
Dyspnea		1 (3.6)
Vascular disorders	n (%) of subjects	1 (3.6)
Orthostatic hypotension		1 (3.6)

TEAE=Treatment-emergent adverse event (with onset on or after Day 1).

Supplemental Table S24. CL-001 Drug Concentration Measurement Times

Part	Cohort	Day	PK Sampling
1	1	-	predose (0 hour) and at 0.5, 1, 1.5, 2, 3, 4, 5, 6, 7, 8, 12, 16, 20, 24, 30, 36, 44, 48, 72, 96, 120, 144, and 168 (D7) h
	2	-	predose (0 hour) and at 0.5, 1, 1.5, 2, 3, 4, 5, 6, 8, 10, 12, 16, 20, 24, 28, 32, 36, 40, 44, 48, 54, 60, 66, 72, 80, 88, 96, 120, 144, and 168 (D7) h
	3	-	predose (0 hour) and at 0.5, 1, 2, 3, 4, 5, 6, 8, 10, 12, 16, 20, 24, 28, 32, 36, 40, 44, 48, 54, 60, 66, 72, 80, 88, 96, 120, 144, 168 (D7), 216 (D9), 312 (D14), 384 (D17), 480 (D21), and 648 (D28) h
	4 (Fasted)	-	predose (0 hour) and at 0.5, 1, 2, 3, 4, 5, 6, 8, 10, 12, 16, 20, 24, 28, 32, 36, 40, 44, 48, 54, 60, 66, 72, 80, 88, 96, 120, 144, 168 (D7), 216 (D9), 312 (D14), 384 (D17), 480 (D21), 648 (D28), 984 (D42), 1320 (D56), and 1660 (D70) h
	4 (Fed)	-	predose (0 hour) and at 0.5, 1, 2, 3, 4, 5, 6, 8, 10, 12, 16, 20, 24, 28, 32, 36, 40, 44, 48, 54, 60, 66, 72, 80, 88, 96, 120, 144, 168 (D7), 216 (D9), 312 (D14), 384 (D17), 480 (D21), and 648 (D28) h
	5	-	predose (0 hour) and at 0.5, 1, 2, 3, 4, 5, 6, 8, 10, 12, 16, 20, 24, 28, 32, 36, 40, 44, 48, 54, 60, 66, 72, 80, 88, 96, 120, 144, 168 (D7), 216 (D9), 312 (D14), 384 (D17), 480 (D21), 648 (D28), 984 (D42), 1320 (D56), and 1660 (D70) h
	6	-	predose (0 hour) and at 0.5, 1, 2, 3, 4, 5, 6, 8, 10, 12, 16, 20, 24, 28, 32, 36, 40, 44, 48, 54, 60, 66, 72, 80, 88, 96, 120, 144, 168 (D7), 216 (D9), 312 (D14), 384 (D17), 480 (D21), 648 (D28), 984 (D42), 1320 (D56), and 1660 (D70) h
	7	-	predose (0 hour) and at 0.5, 1, 2, 3, 4, 5, 6, 8, 10, 12, 16, 20, 24, 28, 32, 36, 40, 44, 48, 54, 60, 66, 72, 80, 88, 96, 120, 144, 168 (D7), and 312 (D14) h
2	1, 2, & 3	1	predose (0 hour) and at 0.5, 1, 2, 3, 4, 5, 6, 8, 10, 12, 16, 20, and 24 hours
		14	predose (0 hour) and at 0.5, 1, 2, 3, 4, 5, 8, 12, 16, 20, 24, 28, 32, 36, 40, 44, 48, 54, 60, 66, 72, 80, 88, 96, 120, 144, 168 (D7), 672 (D29), 1180 (D50), 1680 (D71), 2180 (D92), and 2690 (D113) h<br>Predose trough concentrations were collected daily from Days 2 through 13.

3	1, 2, & 3	-	predose (0 hour) and at 0.5, 1, 2, 3, 4, 5,6, 8, 10, 12, 16, 20, 24, 28, 32, 36, 40, 44, 48, 54, 60, 66, 72, 80, 88, 96, 120, 144, 168 (D7), 216 (D9), and 312 (D14) h

Supplemental Figures

Figure S1 CL-001 MAD (Part 2): Comparing TBAJ-876 Mean Concentration Profiles Day 1 Versus Day 14

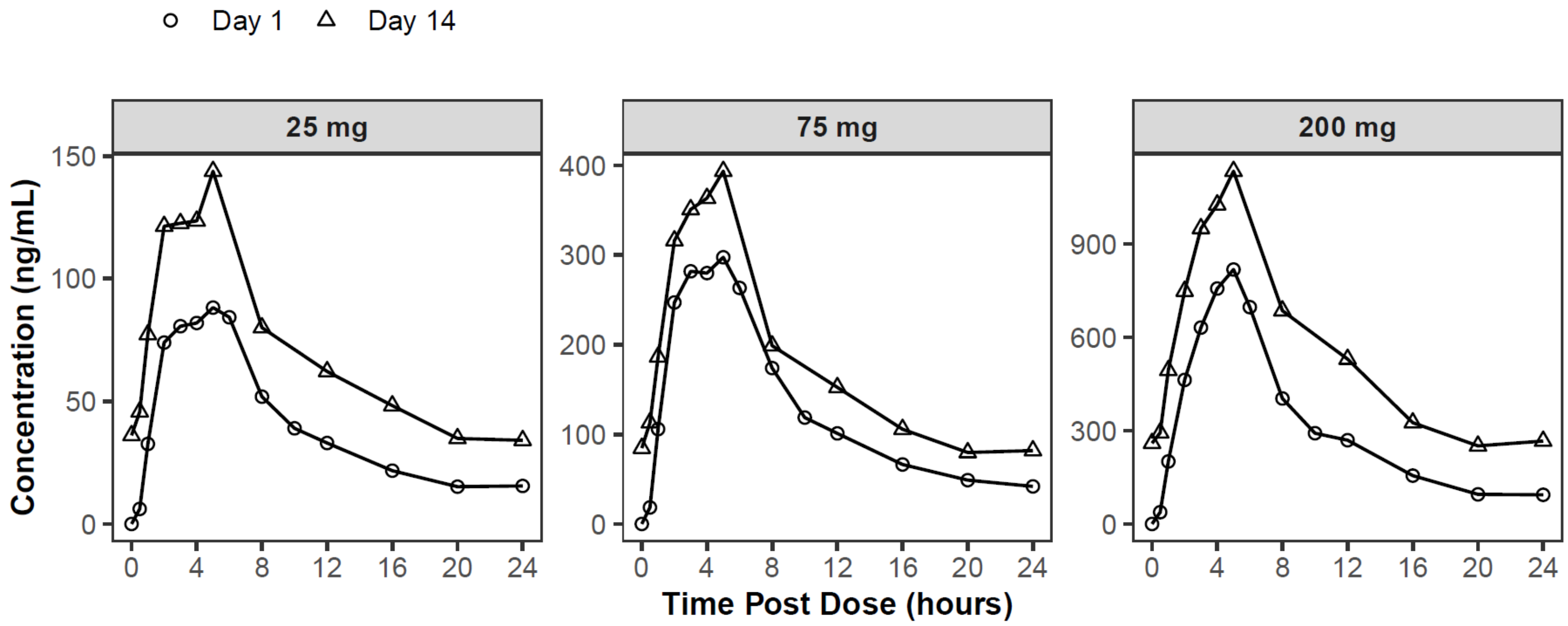

Figure S1: (Supplemental Material) Mean Plasma Concentration-Time Profiles of TBAJ-876 after Single Doses (Day 1) and Multiple Doses (Day 14) of 25 mg (Cohort 1), 75 mg (Cohort 2), and 200 mg (Cohort 3) TBAJ-876 Oral Suspension (Part 2) on Linear Scales

Figure S2 CL-001 MAD (Part 2): Comparing M3 Mean Concentration Profiles Day 1 Versus Day 14

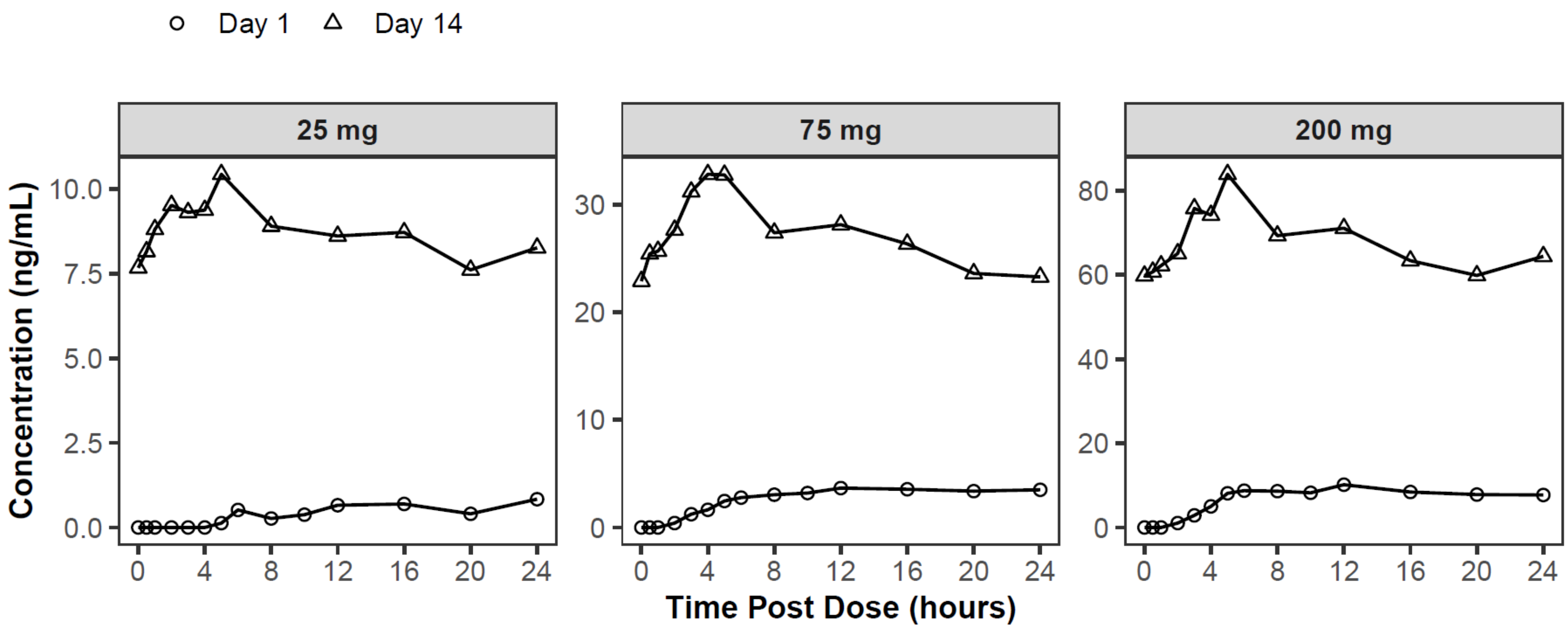

Figure S2: (Supplemental Material) Mean Plasma Concentration-Time Profiles of M3 after Single Doses (Day 1) and Multiple Doses (Day 14) of 25 mg (Cohort 1), 75 mg (Cohort 2), and 200 mg (Cohort 3) TBAJ-876 Oral Suspension (Part 2) on Linear Scales

Figure S3 CL-001 MAD (Part 3): Comparing M2 Mean Concentration Profiles Day 1 Versus Day 14

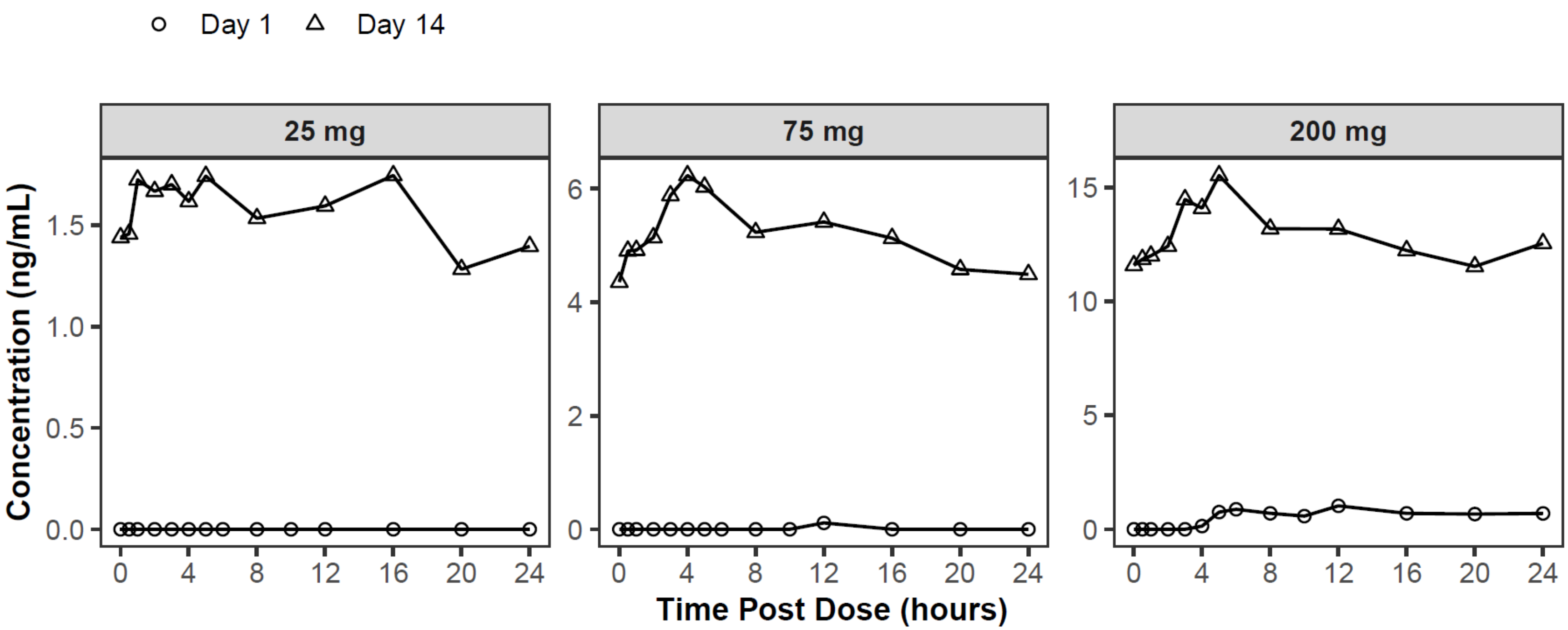

Figure S3: (Supplemental Material) Mean Plasma Concentration-Time Profiles of M2 after Single Doses (Day 1) and Multiple Doses (Day 14) of 25 mg (Cohort 1), 75 mg (Cohort 2), and 200 mg (Cohort 3) TBAJ-876 Oral Suspension (Part 2) on Linear Scales
